# Nutritional adequacy of the EAT-Lancet planetary health diet: cross-sectional analyses of the United Kingdom National Diet and Nutrition Survey

**DOI:** 10.1016/j.ajcnut.2025.11.004

**Published:** 2025-11-13

**Authors:** Vickie S Braithwaite, Solomon A Sowah, Fumiaki Imamura, Nita G Forouhi

**Affiliations:** MRC Epidemiology Unit, Institute of Metabolic Science, University of Cambridge School of Clinical Medicine, Cambridge, United Kingdom

**Keywords:** Planetary health diet, EAT-Lancet reference diet, nutritional adequacy, United Kingdom National Diet and Nutrition Survey, nutritional biomarkers, food diary

## Abstract

**Background:**

The EAT-Lancet planetary health diet (PHD) has been designed to benefit both human and planetary health, but questions remain regarding its nutritional adequacy.

**Objectives:**

This study aimed to assess the nutritional adequacy of the PHD by evaluating diets in the United Kingdom (UK) population and comparing the PHD with the Mediterranean diet.

**Methods:**

Dietary data from participants aged ≥15 y from the nationally representative, serial, cross-sectional UK National Diet and Nutrition Survey (2008–2019) who completed 4-d food diaries (*n* = 9671) were analyzed. Nutritional biomarkers were available in a subset (*n* = 4622). Alignment with the PHD was assessed based on consumption of 14-food components (PHD score range 0–140). Analyses were age-stratified and adjusted for sociodemographic, behavioral, and anthropometric factors.

**Results:**

At the national level, some nutrient intakes were inadequate: for example, ∼50% of the population showed inadequate intakes for iron, zinc, and calcium. The population mean ± standard deviation PHD score was 75.8 ± 13.9 points. Higher alignment with the PHD was associated with a greater likelihood of nutritional adequacy for various nutrients. Positive associations were observed for most vitamins and minerals, with adjusted odds ratios (95% confidence intervals) per 20-point higher PHD score of 2.40 (2.10, 2.76) for iron, 1.26 (1.13, 1.42) for zinc, 1.81 (1.60, 2.04) for calcium, and 3.35 (2.17, 5.16) for vitamin D. No overall association with the PHD was seen for vitamin B12 [0.80 (0.59,1.08)] intake. Either positive or no associations were found between PHD score and nutritional biomarkers. These findings did not differ materially by subgroup or when compared with a Mediterranean-type diet for selected nutrients of concern and their biomarkers.

**Conclusions:**

Nutritional adequacy was either better or unchanged with greater alignment to the PHD, suggesting that the PHD is unlikely to present concerns of nutritional inadequacy at the population level in the UK.

## Introduction

The EAT-Lancet planetary health diet (PHD) was first proposed in 2019 [1], supporting the UN-sustainable development goals and Paris Agreement to optimize human health through nutrition while considering environmental sustainability [2,3]. A PHD is high in fruit and vegetables, whole grains, plant proteins (e.g., from legumes) and contains modest amounts of meat, dairy, added sugars, and starchy vegetables. Studies have described the benefits of a PHD for both planetary and human health [2,3]. Greater alignment with the PHD has been associated with a lower incidence of a number of diseases including type 2 diabetes [[4], [5], [6]], heart failure [7], stroke [8], asthma [9], cancer [10], age-associated cognitive decline [11], and overall mortality [[12], [13], [14]]. However, concern has been raised regarding the potential lack of nutritional adequacy of the PHD, but the existing research evidence is mixed.

In relation to nutritional adequacy, several studies have reported favorable associations between the PHD score and nutrient intake [[15], [16], [17]], adequacy of a PHD compared with the national guidelines (for most nutrients) [18,19], or similar [20] or better adequacy [21,22] of a PHD compared with the standard national diet (for most nutrients). Other studies have cautioned on the ability of a PHD to meet nutritional adequacy for several nutrients in populations, particularly for vitamin B12, vitamin D, calcium, iron, and zinc [[17], [19], [20], [21], [22], [23], [24]]. For example, Beal et al. [23] estimated that only 55% of women of reproductive age (WRA) following an EAT-Lancet diet would reach recommended nutrient intakes for iron. This is of particular concern during pregnancy, when iron requirements increase substantially. Adequate maternal iron intake during pregnancy is critical to prevent iron deficiency anemia and to reduce risk of adverse health outcomes for the mother and infant, such as low birth weight [25].

Many studies to date have described associations between the PHD and nutrient intake [15,26] rather than assessing nutritional adequacy of a PHD and/or contrasting with the nutritional adequacy of the standard current diet. Many have only reported on self-reported nutrient intakes without associated objective nutritional biomarkers [21,26], or have not adjusted for important covariates [22] or have not explored adequacy by population subgroup [22].

The aim of this study was to evaluate the nutritional adequacy of a PHD across nutrients and nutritional biomarkers using data from the United Kingdom (UK) National Diet and Nutrition Survey (2008–2019, NDNS), and to contextualize these results against the nutritional adequacy of the average national diet. Second, we aimed to explore in more detail the nutritional adequacy of a PHD for selected nutrients of concern—namely vitamin B12, vitamin D, calcium, iron, and zinc and associated biomarkers—across different age groups and for WRA. Additional objectives were to contrast the nutritional adequacy, for selected nutrients of concern, of a PHD with a Mediterranean diet (MD; a well-recognized dietary pattern associated with positive health outcomes) [27] and to assess changes in alignment with the PHD over time.

## Methods

### Survey participants

The UK NDNS is a serial survey of the UK general population that collects data on diet, health behaviors, sociodemographic factors and anthropometry [28,29]. The rolling program started in 2008 and has been conducted annually since then. At the time of analysis, the publicly available data were from 2008 to 2019. Approximately 500 adults (aged 19 or over) and 500 children (aged 1.5–18 years (y)) were surveyed annually across the UK, following a multistage clustered stratified design with additional national boosts from Scotland, Wales, and Northern Ireland to ensure representativeness of the UK 4 nations [28]. From a total of 15,655 participants with dietary data available in the data from 2008 to 2019, we excluded 5984 participants aged under 15 y, and the current study sample included 9671 participants (Supplementary Figure 1). Children younger than 15 y were excluded because we adopted the EAT-Lancet diet originally developed based on energy intake requirements of 2500 kcal/day [30] and because the level of energy intake required is higher than the estimated average requirement of energy (EAR) for children [31]. No other exclusion criterion was applied. Children aged 15 y and over were included to allow for analyses based on WRA (15–49 y, as per the WHO definition) [32]. A subset of individuals included in the study also provided blood and urine samples for nutritional biomarker analysis (*n* = 4622).

### Ethics statement

The NDNS program has obtained ethical approval from the National Health Service Health Research Authority (Oxfordshire Research Ethics Committee A, and East of England-Cambridgeshire South Research Ethics Committee). As part of the NDNS fieldwork, questionnaire information was collected with verbal agreement and physical measured and biological samples under written consent from participants (aged ≥16 y) or from parent/carers (for younger aged participants) [28].

### Assessment methods

Detailed descriptions of the dietary assessment method and participant questionnaire are described elsewhere [28]. In brief, for the NDNS study years 1–11, an estimated (nonweighed) food diary was collected in participants over 4 consecutive days. The survey was conducted using a continuous fieldwork model to minimize issues of seasonality. Height and weight were measured and recorded by trained survey staff, and questionnaires were completed to ascertain supplement use, socioeconomic status, equivalized household income tertile, ethnic group (self-reported, from a choice of 5 options: White, Mixed ethnic group, Black or Black British, Asian or Asian British, or any other group), region of residence in the UK [e.g., England: Central/Midlands, England: North, England: South (including London), Scotland, Northern Ireland, and Wales] and smoking status (current smoker, ex-smoker, never a regular smoker). Physical activity was assessed with the recent physical activity questionnaire and expressed as physical activity energy expenditure (PAEE) in kJ/kg/day. From anthropometric measures, BMI (kg/m^2^) was calculated as weight in kilograms divided by the square of the height in meters. Overnight fasted blood and urine (either spot or 24-hour collection) samples were collected among participants who completed their dietary assessment [28].

### Diet scoring

The completed food diaries were coded using a bespoke diet coding program and linked to the NDNS nutrient data bank. An average over the 4 days was used. The participants’ diets were scored based on their alignment with the EAT-Lancet PHD [30], and the MD [33]. Briefly, the PHD scoring reflected consumption levels of the selected dietary components (Supplementary Table 1). A score of between 0 and 10 points was assigned for each of 14 food groups. Specifically, for foods that are encouraged in the PHD, an intake equal to or above the recommended target amount was given 10 points, and nonconsumption was assigned 0 points. These foods included whole grains, fruits, vegetables, nuts, legumes (including beans and soy foods), fish, and unsaturated fat. Conversely, for foods to limit, a score of 10 points was given for consumption equal to or below the recommended amount, and a score of 0 was given for consumption equal to or above the target amount. These foods included starchy vegetables, dairy, red/processed meat, poultry, eggs, fruit juice/added sugars, and saturated fat or trans-fat. Intakes between the minimum and maximum target amounts were assigned proportionately from 0 to 10 points. We then added up the scores for the individual components to obtain a total PHD score for participants, with a possible range from 0 to 140, reflecting the degree of alignment with the PHD.

Diet scoring for the MD was performed using 15 dietary components selected according to the MD pyramid (Supplementary Table 1) [33]. For each of the 15 components, a score from 0 to 1 was assigned according to the participant’s degree of alignment with the recommendation. Different from the PHD, the 0-to-1 point assignment was determined by an absolute level of consumption of each dietary component, and alcohol was included as one of the components and penalized by 0.5 point when overconsumed (Supplementary Table 1).

### Nutritional adequacy assessment

Percentage energy from different macronutrients was calculated for the whole diet. Micronutrient intakes did not include those from dietary supplements as the analysis aimed to assess the nutritional adequacy of the PHD itself rather than from the PHD and dietary supplements. Levels of nutritional intake were compared with the corresponding age- and sex-specific reference nutrient intake (RNI) [31] for micronutrients, vitamins, and protein, EAR for total energy intake, and dietary reference values (DRVs) for fat, fiber, and carbohydrate intake. The RNIs, EAR, and DRVs used in this study are specific to the UK population (Supplementary Tables 2–4) **[**31]. The published NDNS database did not allow for differentiation between animal- and plant-based protein; therefore, total protein was used in this analysis [34].

### Biomarker analysis

Overnight fasted blood samples were collected by nurses/phlebotomists and taken to a laboratory for processing and storage. Blood sample aliquots were used to measure the following nutritional analytes: red blood cell hemolysates riboflavin (measured by erythrocyte glutathione reductase activity coefficient; a marker of riboflavin status), serum folate, serum vitamin B12, serum holotranscobalamin (HoloTC, a marker of biologically active vitamin B12), plasma vitamin C (a marker of fruit and vegetable consumption and of vitamin C), serum 25-hydroxyvitamin D (25OHD) (a marker of vitamin D status), plasma ferritin (an indicator of iron stores), and plasma zinc. Urinary iodine was also measured from a urine sample. Detailed methods for these analytes are described elsewhere [28]. Assay information (coefficients of variation and limits of detection) and biomarker thresholds to identify adequacy of nutritional status used in this study are presented in Supplementary Table 5. As NDNS has no established thresholds for plasma zinc concentrations, this was assessed on a continuous scale and not as a binary outcome.

### Statistical methods

Data analysis was performed in Stata v17 (StataCorp), and Excel v2502 (Microsoft) and R (v4.4.0) were used to produce the figures. For descriptive statistics of the study participants, the analysis sample was analyzed unadjusted. All the further estimations accounted for the sampling weights as specified in the published NDNS dataset and as instructed in the NDNS documents, using “*svy*” commands in Stata, separately for dietary, anthropometry, and biochemistry data [35]. Hypothesis tests were conducted with alpha_2-sided_ = 0.05 each, without adjustment for multiple testing to detect a potential signal of nutritional inadequacy related to the PHD and avoid false-negative findings. Continuous nutrient and biochemical data underwent a logarithmic transformation, and their descriptive statistical data were computed as geometric mean. Participants were split into 5 groups by quintiles of the PHD score. Linear regression models were used to test for trend across the PHD quintiles.

Binary outcomes were defined for each nutrient to identify whether individuals’ intakes were adequate in reference to each nutritional threshold (e.g., RNI/DRV/EAR or biochemical threshold). The percentage of the population reaching or exceeding nutritional adequacy and biomarker thresholds was determined for all nutrients across the whole NDNS population (≥15 y of age). Multiple logistic regressions were modeled to estimate the odds ratios (ORs) of nutritional adequacy for every 20-point higher PHD score (equivalent to ∼1.5 × SD). Three multiple regression models were fitted to control for covariate characteristics: the first model applied no adjustment (crude, unadjusted model). The second model adjusted for age (continuous), sex (males/females), ethnicity (white/non-white), occupation (managerial or professional occupations; intermediate occupations or small employers and own account workers; lower supervisory and technical occupations, semi-routine occupations, or routine occupations; never worked or other; and unanswered/not knowing), equivalized income tertile (ordinal: lowest, middle and highest income tertile), region (Central England/Midlands, North England, South England (including London), Northern Ireland, Scotland, Wales), NDNS survey year (categorical: years 1–11), season (spring, summer, autumn, winter), BMI (kg/m^2^), smoking (current smoker, ex-smoker, never smoker), alcohol consumption (g/day), supplement use (yes or no over the last 12 mo), PAEE (KJ/kg/day), and chronic health conditions (yes or no for 12 mo or more). The third model was further adjusted for total energy intake and was performed.

We conducted complete-case analyses, assuming no substantial bias as demonstrated elsewhere [36]. Detailed analyses of adequacy for selected nutrients and biomarkers of concern (intakes of vitamin B12, vitamin D, calcium, iron, and zinc; and their related biomarkers) were conducted, with stratification by population subgroup: 15–18 y (*n* = 1672), for adults: 19–64 y (*n* = 6136), ≥65 y (*n* = 1863) and for WRA (15–49 y, as per the WHO definition) [32] (*n* = 3350). Nutritional biochemistry was available for a subset of participants (*n* = 4622): 15–18 y (*n* = 623), 19–64 y (*n* = 3105), ≥65 y (*n* = 894), and for WRA (*n* = 1466).

In addition, the likelihood of adequacy for nutrients and associated biomarkers of concern related to the PHD was considered in relation to MD scores after standardizing each score (Z-score) within each age category. This allowed for descriptive comparison of the strengths of associations with nutritional adequacy between the 2 diets, without formal statistical testing.

## Results

### Participant characteristics and diet score

In the study population, the mean age was 44.2 y (range, 15–96), 58% were female, the mean ± SD BMI was 26.8 ± 5.5 kg/m^2^, and 58% of the population had overweight or obesity (BMI ≥25 kg/m^2^). The mean ± SD of the PHD scores was 74.1 ± 13.8 points out of a total possible maximum score of 140 (Table 1). The PHD score was lower among current smokers and higher among nonsmokers, older adults, females, supplement users, individuals with managerial and professional occupations, and among households with higher income compared with lower incomes. The PHD score differed by region (lowest in Northern Ireland: 67 and highest in the South of England: 77). Alignment with the PHD showed an upward trend over the NDNS survey years, and the upward trend was also observed in the MD scores (Supplementary Figure 2).

**TABLE 1:** Characteristics of the population aged 15 y or over by PHD in the total population and by PHD categories: the National Diet and Nutrition Survey (2008–2019) (*n* = 9671)

Characteristics	Total population (*n* = 9671)	Categories of the PHD scores by quintile				
		Q1 (*n* = 1935)	Q2 (*n* = 1934)	Q3 (*n* = 1934)	Q4 (*n* = 1934)	Q5 (*n* = 1934)
Planetary health diet score, range^1^	31.9–122.6	31.9–61.9	62.0–69.8	69.9–77.4	77.5–86.2	86.3–122.6
Mediterranean diet score^1^	6.3 (1.7)	4.7 (1.1)	5.5 (1.1)	6.2 (1.2)	6.9 (1.2)	8.2 (1.3)
Age^1^ (y)	44.2 (20.4)	34.3 (18.8)	40.5 (20.5)	45.6 (20.2)	49.2 (19.8)	51.4 (17.6)
Sex, *n*, % female	5571, 58	838, 43	1046, 54	1115, 58	1255, 65	131, 68
National Statistics socioeconomic classification (NS-SEC3) of occupation of household reference person, *n* %						
Managerial and professional	3872, 40	530, 27	639, 33	745, 39	894, 46	1064, 55
Intermediate	2000, 21	375, 19	413, 21	441, 23	394, 20	337, 19
Routine and manual	3347, 35	914, 47	791, 41	655, 34	571, 30	416, 22
Never worked, other, or no answer	452, 5	116, 6	91, 5	93, 5	75, 4	77, 4
Equivalized household income, *n* %						
Lowest tertile	2758, 29	756, 39	635, 33	566, 29	437, 23	364, 19
Middle tertile	2613, 27	527, 27	549, 28	539, 28	516, 27	482, 25
Highest tertile	2883, 30	378, 20	463, 24	551, 28	680, 35	811, 42
Unknown	1417, 15	274, 14	287, 15	278, 14	301, 16	227, 14
Region, *n* %						
England: central/midlands	1201, 12	233, 12	251, 13	258, 13	238, 12	221, 11
England: north	1722, 18	330, 17	346, 18	340, 18	375, 19	331, 17
England: south	2861, 30	447, 23	490, 25	560, 29	611, 32	753, 39
Northern Ireland	1294, 13	379, 20	295, 15	254, 13	199, 10	167, 9
Scotland	1404, 15	312, 16	279, 14	273, 14	289, 15	251, 13
Wales	1189, 12	234, 12	273, 14	249, 13	222, 11	211, 11
Ethnic group, *n* %						
White	8867, 92	1836, 95	1823, 94	1768, 91	1745, 90	1695, 88
Mixed ethnic group	102, 1	21, 1	14, 0.7	26, 1	20, 1	21, 1
Black or Black British	210, 2	41, 2	33, 2	35, 2	47, 2	54, 3
Asian or Asian British	350,	24, 1	45, 2	75, 4	91, 5	115, 6
Any other group	142, 1	13, 0.7	19, 0.9	30, 2	31, 2	49, 3
Smoking status, *n* %						
Never a regular smoker	5776, 60	1023, 55	1057, 55	1136, 59	1239, 64	1321, 68
Ex-smoker	1948, 20	255, 13	343, 18	426, 22	455, 24	469, 24
Current smoker	1944, 20	657, 34	532, 28	372, 19	240, 12	143, 12
Supplement use (yes), *n* %	3086, 32	368, 19	503, 26	608, 31	702, 36	905, 47
Alcohol consumption (g/d)^1^	10.1 (19.8)	10.5 (22.7)	10.5 (23.1)	10.6 (20.8)	9.6 (16.1)	9.3 (14.9)
Physical activity energy expenditure^1^ (kJ/kg/d)	31.9 (32.3)	36.7 (37.8)	32.0 (32.7)	30.9 (32.4)	29.4 (28.5)	30.8 (28.8)
Anthropometry^1^						
Weight (kg)	75.4 (17.0)	78.6 (18.6)	76.2 (17.9)	76.8 (17.0)	75.0 (16.0)	73.5 (15.5)
Height (m)	1.67 (0.09)	1.69 (0.09)	1.68 (0.09)	1.68 (0.09)	1.66 (0.09)	1.66 (0.09)
BMI (kg/m^2^)	26.8 (5.5)	26.1 (5.9)	27.0 (5.9)	27.3 (5.6)	27.1 (5.2)	26.5 (4.9)
Adiposity status (BMI), *n* %						
Underweight <18.5 kg/m^2^	255, 3	96, 5	56, 3	50, 3	33, 2	20, 1
Normal weight 18.5–25 kg/m^2^	3484, 39	794, 44	683, 39	600, 34	619, 35	788, 43
Overweight >25–30 kg/m^2^	2965, 33	506, 28	549, 31	610, 34	661, 37	639, 35
Obese >30 kg/m^2^	2246, 25	413, 28	484, 27	516, 29	463, 26	370, 20

Abbreviations: PHD, planetary health diet.

1 Except for the PHD, mean (SDs) are reported. For categorial variables *n*, %. Mean ± SD of the PHD scores was 74.1 ± 13.8 points. To describe the study sample, crude statistics are presented without estimations accounting for survey design and sampling weights.

### Nutritional adequacy of the average UK diet and of the PHD

At the UK level, on average, ∼76% of the study population had adequate protein intake, 6% of the population achieved the DRV for fiber intake, 29% for total carbohydrate intake, 13% for free sugars, 60% for total fat, and 36% for saturated fat. Furthermore, 13% of the population achieved the EAR for energy intake. Niacin was the only micronutrient with population adequacy (>97.5% of the population reaching the RNI). Adequacy of intake for other vitamins was 96% for vitamin B12, 90% for thiamine, 80% for vitamin B6, 77% for vitamin C, 70% for riboflavin, 64% for folate, 55% for vitamin A, and 1.3% for vitamin D. The adequacy of mineral intake was 97% for phosphorous, 76% for chloride, 73% for sodium, 59% for calcium, 53% for iodine, 50% for iron and zinc, 38% for copper, 32% for magnesium, 19% for potassium and 14% for selenium (Table 2 and Supplementary Table 6).

**TABLE 2:** Estimated proportions of individuals aged ≥15 y old reaching the nutritional guidelines for nutrient intake in the United Kingdom NDNS 2008–2019 (*n* = 9671)^1^

Nutrients	Total	Categories of the PHD scores split by quintiles					*P-*trend^2^
		Q1	Q2	Q3	Q4	Q5	
Energy, EAR%	13.4	16.5	12.2	11.9	12.7	14.0	0.187
Protein, RNI%	75.7	75.6	73.8	73.1	76.9	78.6	0.746
Fiber, g. DRV%	6.4	1.5	1.9	3.0	5.3	17.3	<0.001
Carbohydrate, DRV%	28.7	27.7	25.9	27.4	29.8	31.6	<0.001
Free sugars, DRV%	12.6	6.0	9.5	10.2	13.4	21.0	<0.001
Total fat, DRV%^3^	60.3	58.7	57.6	59.5	63.8	61.2	<0.001
Saturated fat, DRV%^3^	35.6	30.1	28.9	31.6	36.9	46.9	<0.001
Thiamine, RNI%	89.5	84.2	86.4	87.8	92.0	94.8	<0.001
Riboflavin, RNI%	70.0	62.4	67.9	68.1	75.3	74.1	<0.001
Niacin, RNI%	98.5	98.6	97.7	98.0	98.2	99.7	0.004
Vitamin B6, RNI%	79.9	78.7	77.4	76.7	81.8	83.9	<0.001
Vitamin B12, RNI%	96.2	97.0	96.5	95.5	95.9	96.1	0.242
Folate, RNI%	63.6	50.8	53.4	60.5	68.8	78.7	<0.001
Vitamin C, RNI%	77.3	63.6	66.0	74.8	83.5	92.8	<0.001
Vitamin A (RE). RNI%	55.3	42.5	47.6	52.7	60.7	68.1	<0.001
Vitamin D, RNI%	1.3	0.0	0.8	1.0	1.4	2.6	<0.001
Calcium, RNI%	58.5	51.5	56.1	56.6	61.6	64.4	<0.001
Phosphorus, RNI%	97.4	96.7	96.4	97.0	97.6	98.8	<0.001
Magnesium, RNI%	32.1	16.7	20.1	25.1	37.0	54.1	<0.001
Sodium, RNI%	73.2	80.4	76.6	71.8	73.8	65.8	0.051
Sodium, DRV%^3^	68.0	56.0	65.4	68.5	69.9	76.8	0.124
Potassium, RNI%	18.6	15.1	14.2	16.0	19.1	26.5	<0.001
Chloride, RNI%	76.4	80.9	77.5	74.8	76.7	73.3	0.340
Iron, RNI%	49.9	41.4	42.7	48.4	53.4	59.7	<0.001
Zinc, RNI%	49.9	45.1	50.4	45.6	51.4	55.4	0.002
Copper, RNI%	38.3	26.7	28.4	33.2	42.1	55.3	<0.001
Selenium, RNI%	13.5	7.1	8.1	10.9	15.5	22.6	<0.001
Iodine, RNI%	53.1	42.8	50.4	51.8	57.3	59.9	<0.001

Abbreviations: DRV, dietary reference value; EAR, estimated average requirement; NDNS, National Diet and Nutrition Survey; RE, retinol equivalent; RNI, reference nutrient intake.

1 Proportions of those meeting each nutritional criterion were estimated, with the survey design and sampling weights accounted for. Mean ± SD of the PHD was 75.8 ± 13.9 points. Five quintile categories had the ranges of the PHD scores of <62.0, 62.0–69.8, 69.9–77.4, 77.5–86.2, and ≥86.3 points, respectively. Detailed results are presented in Supplementary Table 6.

2 *P* values for trend across the quintile categories were computed using survey-weighted logistic regression models adjusted for age, sex, ethnicity, occupation, income, region, survey year, season, BMI, smoking, alcohol consumption, supplement use, physical activity energy expenditure, chronic health conditions, and energy intake.

3 For total fat, saturated fat, free sugars, and sodium DRV, the percentage refers to those who consumed less than the DRV: for example, the higher the percentage, the greater the number not exceeding the nutritional guideline.

The mean PHD score was 75.8 points when survey design and sampling weights were applied. No nutrient indicated a higher prevalence of inadequacy when the PHD score was higher. Greater alignment with the PHD was associated with a greater likelihood of nutritional adequacy for a range of dietary factors. The adjusted OR [95% confidence intervals (CIs)] for nutritional adequacy per 20-point higher PHD score were as follows: fiber, 9.93 (7.70, 12.81); carbohydrate, 1.32 (1.17, 1.48); free sugars, 2.34 (1.98, 2.76); total fat, 1.17 (1.06, 1.30); saturated fat, 1.88 (1.69, 2.08); thiamine, 2.37 (1.96, 2.87); riboflavin, 1.42 (1.25, 1.62); niacin, 2.28 (1.54, 3.39); vitamin B6, 1.41 (1.22, 1.62); folate, 3.03 (2.65, 3.46); vitamin C, 2.85 (2.49, 3.25); vitamin A, 1.68 (1.50, 1.87); vitamin D, 3.35 (2.17, 5.16); calcium, 1.81 (1.60, 2.04); phosphorous, 2.83 (1.92, 4.17); magnesium, 8.59 (7.20, 10.24); potassium, 2.33 (1.99, 2.72); iron, 2.40 (2.10, 2.76); zinc, 1.26 (1.13, 1.42); copper, 3.93 (3.45, 4.48); selenium, 2.23 (1.91, 2.60); and iodine, 1.59 (1.42, 1.77). The PHD was not associated with adequacy for energy, 0.90 (0.76, 1.07); protein, 0.98 (0.85, 1.14); vitamin B12, 0.80 (0.59, 1.08); or chloride, 1.06 (0.91, 1.23) intakes in the energy-adjusted models (model 3) (Table 2 and Supplementary Table 6). In nonenergy-adjusted models (model 2), higher alignment with the PHD was associated with lower vitamin B12 intake [0.72 (0.57, 0.92), per 20-point higher PHD score].

When consuming the usual diet, over 90% of the population had nutritional biomarker levels over the biochemical thresholds, including circulating vitamin B12, HoloTC, and ferritin. Approximately 83% had a 25OHD concentration over the biochemical threshold, 61% for folate, 57% for urinary iodine and 40% for riboflavin. Across the PHD score the likelihood of reaching the biochemical thresholds for nutritional biomarkers did not differ significantly, with the exception of higher 25OHD and folate concentrations: the adjusted ORs (95% CIs) were 1.46 (1.20, 1.78) and 2.17 (1.88, 2.50) per 20-point higher PHD score, respectively (Table 3 and Supplementary Table 7).

**TABLE 3:** Estimated proportions of individuals aged ≥15 y old reaching the biochemical thresholds for nutritional biomarkers in the United Kingdom National Diet and Nutrition Survey (NDNS) 2008–2019 (*n* = 4622)^1^

Nutritional biomarker^2^	Total	Categories of the PHD scores split by quintiles					*P*-trend^3^
		Q1	Q2	Q3	Q4	Q5	
Vitamin B12, %	95.3	96.2	95.7	94.7	95.9	94.4	0.119
HoloTC, %	93.7	91.0	95.0	92.9	95.3	93.6	0.255
25-OHD, %	83.4	76.3	83.6	81.2	84.3	88.9	0.001
Riboflavin (EGRAC), %	39.7	28.6	34.7	44.1	42.3	44.5	0.296
Folate, %	61.4	38.2	49.6	60.9	69.5	78.3	<0.001
Ferritin, %	92.4	94.3	92.5	91.2	92.3	92.0	0.273
Iodine, %	57.3	54.7	61.8	56.3	61.2	53.6	0.132

Abbreviations: 25OHD, 25-hydroxyvitamin D; EGRAC, erythrocyte glutathione reductase activity coefficient; HoloTC, Holotranscobalamin; NDNS, National Diet and Nutrition Survey; PHD, planetary health diet.

1 Proportions of those meeting each nutritional criterion were estimated, with the survey design and sampling weights accounted for. Mean ± SD of the PHD was 75.8 ± 13.9 points. Five quintile categories had the ranges of the PHD scores of <62.0, 62.0–69.8, 69.9–77.4, 77.5–86.2, and ≥86.3 points, respectively. Detailed results are presented in Supplementary Table 7.

2 The biomarker thresholds and assay information are available in the Supplementary Table 5. Folate was the sum of 6 folate derivatives; ferritin for iron storage.

3 *P* values for trend across the quintile categories were computed using survey-weighted logistic regression models adjusted for age, sex, ethnicity, occupation, income, region, survey year, season, BMI, smoking, alcohol consumption, supplement use, physical activity energy expenditure, chronic health conditions, and energy intake.

### Nutritional adequacy by population subgroup for selected nutrients of concern

In the usual UK diet, adequacy of vitamin B12 intake was high across all population subgroups (≥94% achieving the RNI) but was lower for vitamin D intake (adolescents: 0.3%, 19–64 y olds: 1.3%, ≥65 y: 1.3%, and WRA: 0.8%), calcium (adolescents: 27%, 19–64 y olds: 60%, ≥65 y: 63% and WRA: 46%), iron (adolescents: 21%, 19–64 y olds: 49%, ≥65 y: 60% and WRA: 4.8%), and zinc (adolescents: 37%, 19–64 y olds: 52%, ≥65 y: 46% and WRA: 53%) (Supplementary tables 8–11).

**FIGURE 1:**
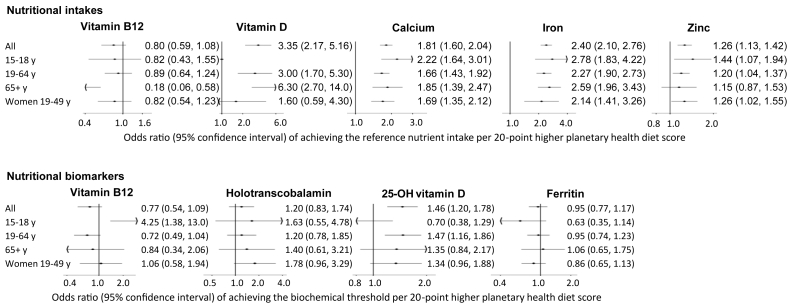
Associations of PHD with the likelihood of achieving the reference nutrient intake and biochemical threshold. Adjusted ORs (95% confidence interval) per 20-point higher PHD are presented, estimated from multivariable-adjusted logistic regression controlling for age, sex, ethnicity, occupation, income, region, survey year, season, BMI, smoking, alcohol consumption, supplement use, physical activity energy expenditure, chronic health conditions, and total energy intake. An OR >1 represents a positive association of the PHD with nutritional adequacy based on the biomarker. The nutrients of concern are presented, based on dietary intakes (top) and biomarkers (bottom): holotranscobalamin for vitamin B12 status; 25-OH for vitamin D, and ferritin for iron status. For vitamin D intake in 15–18 y olds, there were insufficient participants reaching the reference nutrient intake and so logistic regression models were not fitted. Specific cutoffs and results for the other nutrients and markers are available in Supplementary Materials. OR, odds ratio; PHD, planetary health diet.

Across PHD score quintiles, the nutritional adequacy for calcium and iron intakes was higher for all population subgroups (Figure 1, Supplementary Tables 8–11). For example, for WRA, the likelihood of reaching nutritional adequacy for iron and calcium intakes was 2.14 (1.41, 3.26) and 1.69 (1.35, 2.12) per 20-point higher PHD score, respectively (Supplementary Table 11). For adolescents, 19–64 y olds and WRA, nutritional adequacy for zinc was positively associated with higher PHD score [Figure 1, 1.44 (1.07, 1.94), 1.20 (1.04, 1.37), and 1.26 (1.02, 1.55), respectively]. Nutritional adequacy for vitamin D intake was higher across PHD score quintiles for 19–64 y olds and ≥65 y olds, but no association was seen for adolescents or WRA (Figure 1). For dietary vitamin B12, nutritional adequacy was not associated with PHD score in any of the population subgroups with the exception of ≥65 y olds where there was an inverse association in energy-adjusted models [0.18 (0.06, 0.58), Figure 1] but not when energy was not adjusted for [0.38 (0.12, 1.23), Supplementary Table 10]. In nonenergy-adjusted models, nutritional adequacy for vitamin B12 intake was lower for 19–64 y olds [0.77 (0.59, 0.99) and WRA (0.72 (0.53, 0.98)], but this was not significant in model 3 (when energy intake was adjusted for, Supplementary Table 9 and 11).

In the usual UK diet, 95% of participants overall achieved the biomarker threshold for vitamin B12 concentrations, and 94% for HoloTC and these percentages were similar across population subgroups (Table 3, Supplementary Tables 7, 12–15). The percentage of participants reaching the biomarker thresholds for ferritin and 25OHD concentrations varied more between population subgroups (ferritin: adolescents: 82%, 19–64 y olds: 92%, ≥65 y: 96%, and WRA: 82%; 25OHD: adolescents: 79%, 19–64 y olds: 83%, ≥65 y: 86%, and WRA: 83%).

For every 20-point higher PHD score in adolescents, the likelihood of reaching vitamin B12 concentration biomarker thresholds was higher [adjusted OR 4.25 (95% CI: 1.38, 13.05)], but no association was observed for other biomarkers (Figure 1, Supplementary Table 12). The likelihood of reaching the biomarker thresholds among 19–64 y olds did not differ across the PHD score with the exception of 25OHD concentrations [1.47 (1.16, 1.86)] where the adjusted likelihood was greater per 20-point higher PHD score (Figure 1, Supplementary Table 13). The likelihood of reaching the biomarker thresholds in WRA did not differ across PHD score quintiles, except for HoloTC concentrations, where the likelihood was higher across PHD quintiles (Supplementary Table 15). The likelihood of reaching the biomarker thresholds in ≥65 y olds did not differ across PHD scores for any of the biomarkers (Supplementary Table 14).

### Nutritional adequacy of the PHD in contrast with the MD scores for selected nutrients of concern

Overall, higher PHD and MD scores were both associated with higher odds of nutritional adequacy for iron, zinc, calcium, and vitamin D intakes with similar effect sizes for both diets (Supplementary Figure 3). The MD scores were positively associated with the likelihood of adequacy for vitamin B12 intake, but no significant result was seen for the PHD score. In the analysis of the nutritional biomarkers, higher PHD and MD scores were both associated with a higher likelihood of the adequacy of vitamin D status based on 25OHD concentrations (Supplementary Figure 4). No association was observed with the other biomarkers**.**

## Discussion

Using the nationally representative UK NDNS, we found overall that higher alignment with the PHD was unlikely to pose the risk of being inadequate in nutrient intakes and status. Our findings and interpretations are broadly consistent with studies from France [21], United States [22], Italy [20], and Australia [17] and the original EAT-Lancet commission [1] but differs from a study from the Netherlands that predicted calcium and vitamin D inadequacies in those following a modeled theoretical PHD [24]. We identified no evidence of the associations between higher PHD score and adequacy for energy, protein, vitamin B12, sodium or chloride intakes in the most adjusted models. This contrasts with findings from the DONALD cohort, which reported modestly lower total protein intake (as a percentage of energy intake) among those in the highest PHD tertile compared with those in the lowest PHD tertile (13.5 % compared with 14.5 % respectively) [15]. Study design and demographic differences may partly explain this discrepancy; the DONALD study (n = 298), Dortmund, Germany, with a median age of 18 y applied a simple method to derive the PHD score. Moreover, regional differences in the availability of plant-based protein sources should also be considered. In keeping with our findings, a Brazilian study also found no association between total protein intake and PHD index [37]. In contrast, other studies have not explored the association between the PHD and total protein intake [22,23].

Nutritional adequacy for iron in the NDNS participants on the usual reported diet was lower in WRA (5% reaching the RNI) and adolescents (21% reaching the RNI) compared with the overall population (50% reaching the RNI). We observed higher nutritional adequacy for iron across higher PHD scores for WRA and adolescents as well as the overall survey population, contrary to findings by Beal et al. [23], but consistent with the EAT-Lancet commission report [1] and others [22,37]. The proportion reaching the biochemical threshold for serum ferritin was lower in WRA and adolescents compared with the entire NDNS population but the likelihood of meeting the ferritin biochemical threshold did not differ by the PHD score overall, nor by any population subgroup in adjusted models.

Nutritional adequacy was particularly low for vitamin D intake in the average UK diet with only 1.3% of the population reaching the RNI. Acknowledging the limited role of the diet in acheiving adequate vitamin D status, underpins the UK government guidelines, which recommend sun exposure and vitamin D supplementation as strategies to improve adequacy [38]. However, we noted that both vitamin D intake and status (25OHD concentrations) were higher across quintiles of the PHD score, with 83% of participants reaching the biochemical threshold for 25OHD (>25 nmol/L) overall.

The EAT-Lancet Commission and others acknowledged the potential inadequacy for vitamin B12 among those who consume plant-based diets [1] or the PHD [1,23,39], particularly for older adults [40]. Many dietary guidelines recommend considering vitamin B12 supplements among vegans or vegetarians. For instance, in the UK, it is highlighted that vitamin B12 supplements may be required for those following a plant-based diet without sources of vitamin B12 [41] (e.g., meat, eggs, dairy, fish, or foods fortified with vitamin B12). The US guidance also notes that many older adults (>50 y of age) should get vitamin B12 from fortified foods or dietary supplements because of their inability to absorb vitamin B12 [42]. In the UK population, we found that for older adults, higher PHD scores were associated with lower vitamin B12 intakes. On the other hand, no detectable differences were observed across the PHD score in circulating vitamin B12 or HoloTC concentrations in older adults or overall.

In general, alignment with the PHD was moderate in the NDNS population (median: 73, range of 32–123 out of a maximum score of 140) and not markedly different from the US national survey (median PHD score of 67 in adults) [22]. The PHD score increased over years, again in keeping with the US findings [22], and related to the MD scores, as reported from the European HELENA study [16]. Additionally, we found no material difference in the likelihood of reaching nutritional adequacy or biochemical thresholds between higher PHD and MD scores, except for vitamin B12 intake which was positively associated with MD score but showed no significant association with PHD score. Demographic characteristics associated with the PHD were consistent with other studies [15,16,26]: higher in older adults, females than males, and high in those at higher socioeconomic positions.

Public health implications from our findings deserve discussion. We have identified no evidence for increased nutritional inadequacy upon the potential improvement in alignment to the PHD in a UK population. Given the urgent global need to shift toward sustainable dietary patterns, our findings are positive and promising. From a policy perspective, the findings provide reassurance that strategies to promote more healthy and sustainable eating—through dietary guidelines and food system incentives—are unlikely to compromise nutritional adequacy. Considering our study limitations (discussed below) and many related variables not studied here, such as greenhouse gas emissions, food affordability, cultural acceptability, and clinical outcomes, future monitoring in the national survey or specific targeted survey (e.g., low socioeconomic groups) should be conducted to ensure the population-level nutritional adequacy without socioeconomic and health disparities. Together, these considerations highlight that although our findings are reassuring, continued vigilance and supportive policy measures will be essential to secure both health and sustainability benefits of the PHD.

This study presented nationally representative results from the UK, analyzing both subjective and objective dietary measures, accounting for sociodemographic and behavioral factors in the analysis, and evaluating the MD score. None of the previous studies included all of these elements. Our study limitations included our inability to assess the bioavailability of iron or zinc by accounting for phytate intakes. One observational French study and a simulation study from food composition data accounted for iron and zinc bioavailability and estimated inadequacy of bioavailable iron and zinc intakes associated with greater PHD alignment [21,23] Our evaluation of iron (ferritin) and zinc intakes and biomarkers found no evidence of inadequacy across the PHD in the population overall. Our study was also limited in evaluating protein adequacy. We assessed the quantity of total protein intake, which did not vary by degree of alignment with the PHD, but we were unable to assess protein quality or protein sources (e.g., animal and plant products) because of the constraints in the published NDNS datasets. Future work would be required to create the variable in the food composition and consumption data to enable such analyses. An additional limitation is, similarly to other surveys [22], that as food diaries were collected over 4 consecutive days, they may not reflect habitual diets over time. Moreover, the data could be inaccurate against the volunteers’ true habitual diets, as they might behave differently when diet-recording (reactivity bias). The current cross-sectional design limited our ability to assess causality and rule out reverse causality. Individuals with poorer health status may have recently adopted healthier diets (e.g., more aligned to a PHD) after a health event or after receiving health advice. However, this seemed unlikely given the demographic and anthropometric profiles associated with greater alignment with the PHD. Nutritional adequacy could not be assessed for the maximal possible PHD score (140), which was unsurprisingly not reached at the population level. However, our nutritional adequacy assessment covered score points of 86 to 123 in the highest quintile of the PHD. Finally, we acknowledge that as we have used UK nutritional recommendations, our findings may not be generalizable to other countries.

In conclusion, nutritional adequacy was either better or unchanged with greater alignment to the PHD, suggesting that the PHD is unlikely to present concerns of nutritional inadequacy at the population level in the UK. Our findings strengthen the case for considering the PHD as a framework to guide tailored recommendations that safeguard nutritional adequacy across diverse population groups while supporting both human and planetary health in a sustainable way.

## Author contributions

The authors’ responsibilities were as follows – VSB: conducted the analysis, performed the literature search, and wrote the original draft of the manuscript with supervision from NGF; SAS, FI: curated the data, calculated diet scores and provided statistical input; and all authors: provided input into subsequent reviews of the manuscript.

## Data availability

The data used in this study are publicly available.

## Funding

The United Kingdom National Diet and Nutrition Survey is funded by United Kingdom Government: Office for Health Improvement and Disparities (Department of Health and Social Care) and the Food Standards Agency. The survey is currently delivered by NatCen Social Research and the MRC Epidemiology Unit at the University of Cambridge. NGF, SS, and FI were supported by core MRC Epidemiology funding (MC-UU_00006/3). NGF acknowledges the National Institute for Health and Care Research (NIHR) Cambridge Biomedical Research Centre Theme on Nutrition, Obesity, Metabolism and Nutrition (NIHR203312). She is an NIHR Senior Investigator (NIHR202397). VSB is funded through the NHS England School of Public Health, East of England. The views expressed are those of the authors and not necessarily those of the Department of Health and Social Care or the funders.

## Conflict of interest

NGF, SAS, and FI report financial support was provided by Medical Research Council (MRC) Epidemiology Unit (MC_UU_00006/3). NGF reports financial support was provided by National Institute for Health and Care Research (NIHR) Cambridge Biomedical Research Centre (BRC) theme on Nutrition, Obesity, Metabolism and Endocrinology (NIHR203312). NGF reports financial support was provided by NIHR Senior Investigator (NIHR202397). VSB reports financial support was provided by NHS Health Education England East of England. NGF reports a relationship with EAT-Lancet Commission on Healthy, Sustainable, and Just Food Systems that includes: travel reimbursement. NGF reports a relationship with World Health Organization Guideline Development Committee on optimal intake of animal-source foods. that includes: travel reimbursement. The other authors declare that they have no known competing financial interests or personal relationships that could have appeared to influence the work reported in this article.
