# Supplementary material for: Nutritional adequacy of the EAT-Lancet planetary health diet: cross-sectional analyses of the United Kingdom National Diet and Nutrition Survey
Source: Am J Clin Nutr. 2025 Nov 13;123(1):101113. doi: 10.1016/j.ajcnut.2025.11.004 (PMC12851877; doi:10.1016/j.ajcnut.2025.11.004)
Supplement: Multimedia component 1 [file mmc1.docx]

**Supplementary Materials**

Nutritional adequacy of the EAT-Lancet planetary health diet: cross-sectional analyses of the United Kingdom National Diet and Nutrition Survey.

Vickie S Braithwaite (PhD), Solomon A Sowah (PhD), Fumiaki Imamura (PhD), Professor Nita G Forouhi (FMedSci)

**Affiliation for all authors**

MRC Epidemiology Unit,

University of Cambridge School of Clinical Medicine,

Institute of Metabolic Science,

Cambridge Biomedical Campus,

Cambridge, CB2 0QQ,

United Kingdom

**Contents**

[Supplementary Figure 1: Flow-chart of the participant selection: the UK National Diet and Nutrition Survey, 2008-2019. 3](#_Toc215564995)

[Supplementary Figure 2. Trends of planetary health diet score and Mediterranean diet score over time and by age group using the UK National Diet and Nutrition Survey 2008-2019. 4](#_Toc215564996)

[Supplementary Figure 3. Comparison between adjusted odds ratios (95% Confidence Interval) of meeting the reference nutrient intake with higher standardized planetary health diet score and standardized Mediterranean diet score for key selected nutrients by age group. 5](#_Toc215564997)

[Supplementary Figure 4. Comparison between adjusted odds ratios (95% Confidence Interval) of meeting the biochemical threshold with increasing standardized planetary health diet score and standardized Mediterranean diet score for selected nutrients by age group. 6](#_Toc215564998)

[Supplementary Table 1. Planetary health diet and Mediterranean diet scores based on scoring systems described elsewhere. ^1, 2^ 7](#_Toc215564999)

[Supplementary Table 2. Reference Nutrient Intakes by age and sex for dietary vitamins in the United Kingdom.^3^ 8](#_Toc215565000)

[Supplementary Table 3. Reference Nutrient Intakes by age and sex for dietary minerals and dietary reference value (DRV) for sodium intake in the United Kingdom.^3^. 8](#_Toc215565001)

[Supplementary Table 4. Estimated average reference (EAR) by age and sex for Energy intake, Reference nutrient intake (RNI) for protein, and dietary reference values for fiber, total carbohydrate, free sugar, fat and saturated fat intakes per day for macronutrients in the United Kingdom.^3^ 9](#_Toc215565002)

[Supplementary Table 5. Nutritional biomarker assays and cut-offs as used in the National Diet and Nutrition Survey in the United Kingdom.*^4, 5^ 10](#_Toc215565003)

[Supplementary Table 6. Nutrient intake per day in participants ≥15 years in the UK National Diet and Nutrition Survey 2008-2019 (n=9671). 11](#_Toc215565004)

[Supplementary Table 7. Nutritional biomarker status for participants aged ≥15 years in the UK National Diet and Nutrition Survey 2008-2019 (n=4622). 15](#_Toc215565005)

[Supplementary Table 8. Intake of selected nutrients per day for adolescents aged 15-18 year olds in the UK National Diet and Nutrition Survey 2008-2019 (n=1672). 17](#_Toc215565006)

[Supplementary Table 9. Intake of selected nutrients per day for adults aged 19-64 years of age in the UK National Diet and Nutrition Survey 2008-2019 (n=6136). 18](#_Toc215565007)

[Supplementary Table 10. Intake of selected nutrients per day for adults aged 65 years of age and over in the UK National Diet and Nutrition Survey 2008-2019 (n=1863). 19](#_Toc215565008)

[Supplementary Table 11. Intake of selected nutrients per day for women of reproductive age (15-49 years) in the UK National Diet and Nutrition Survey 2008-2019 (n=3350). 20](#_Toc215565009)

[Supplementary Table 12. Nutritional biomarker status of selected nutrients in adolescents aged 15-18 years in the UK National Diet and Nutrition Survey 2008-2019 (n=632). 21](#_Toc215565010)

[Supplementary Table 13. Nutritional biomarker status of selected nutrients in participants aged 19-64 years in the UK National Diet and Nutrition Survey 2008-2019 (n=3105). 22](#_Toc215565011)

[Supplementary Table 14. Nutritional biomarker status of selected nutrients in participants aged 65 years and above in the UK National Diet and Nutrition Survey 2008-2019 (n=894). 23](#_Toc215565012)

[Supplementary Table 15. Nutritional biomarker status of selected nutrients in women of reproductive age (15-49 years) in the UK National Diet and Nutrition Survey 2008-2019 (n=1466). 24](#_Toc215565013)

[References of supplementary materials 25](#_Toc215565014)

**
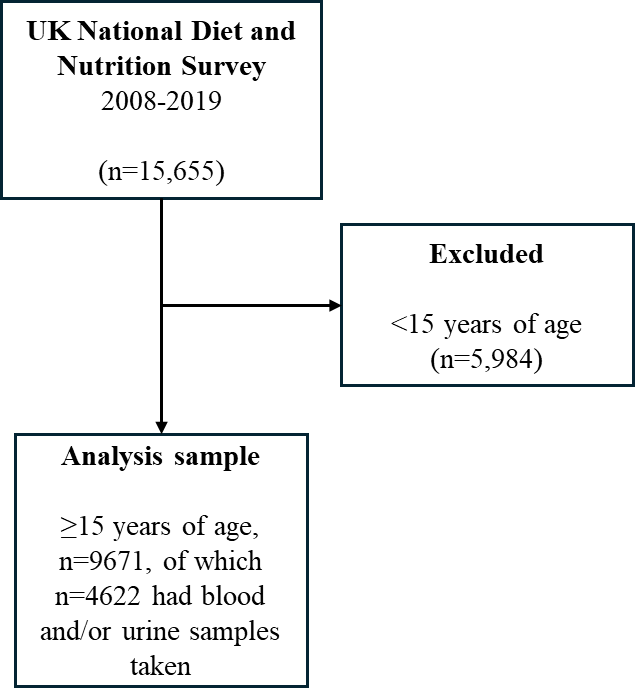
**

# Supplementary Figure 1: Flow-chart of the participant selection: the UK National Diet and Nutrition Survey, 2008-2019.

# **
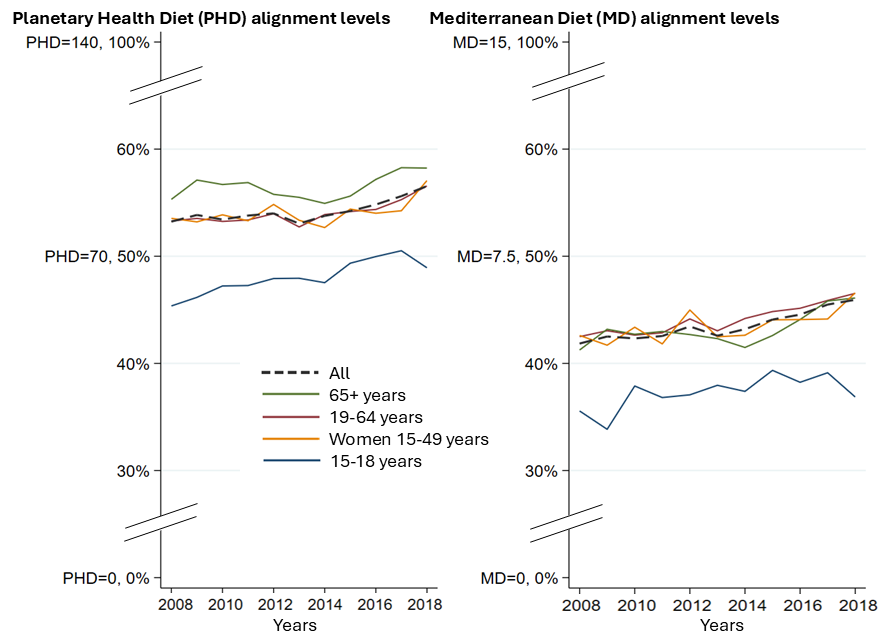
Supplementary Figure 2.** **Trends of planetary health diet score and Mediterranean diet score over time and by age group using the UK National Diet and Nutrition Survey 2008-2019**.

Average percentages of maximum alignment are presented over the survey years (i.e. out of a maximum 140 for the planetary health diet and a maximum of 15 for the Mediterranean diet score). Both scores increased over the NDNS survey years, according to the results from unadjusted linear regression models regressing each diet score on time in years and covariates: β-coefficient per year of 0.33 (95% confidence interval, 0.21, 0.46) for the planetary health diet score; and 0.05 (0.04, 0.07) for the Mediterranean diet score; and in the adjusted models, 0.33 (0.17, 0.49) and 0.05 (0.03, 0.07), respectively (adjusted for age, sex, ethnicity, occupation, income, region, survey year, season, body-mass index, smoking, alcohol consumption, supplement use, physical activity energy expenditure, chronic health conditions and total energy intake).

MD: Mediterranean diet. NDNS: National Diet and Nutrition Survey. PHD: Planetary health diet.


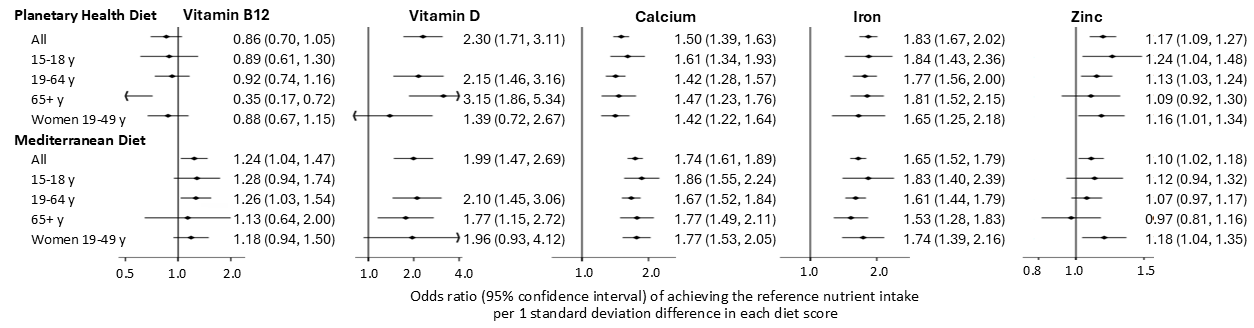


# Supplementary Figure 3. Comparison between adjusted odds ratios (95% Confidence Interval) of meeting the reference nutrient intake with higher standardized planetary health diet score and standardized Mediterranean diet score for key selected nutrients by age group.

Vitamin B12 (µg/day), Vitamin D (µg/day), Calcium (mg/day), Iron (mg/day), and Zinc (mg/day). Logistic regression models adjusted for age, sex, ethnicity, occupation, income, region, survey year, season, body-mass index, smoking, alcohol consumption, supplement use, physical activity energy expenditure, chronic health conditions and total energy intake. Standardized diet scores were calculated by subtracting the population mean from the raw score and then dividing by the population standard deviation for each diet and each age group in turn. An odds ratio of 1 represents no association, <1 represents an inverse association and >1 represents a positive association between the diet score and the likelihood of reaching the reference nutrient intake. For vitamin D intake in 15-18 year olds, there were insufficient participants reaching the reference nutrient intake and so logistic regression models were not fitted. Models were conducted using the survey design and sampling weights accounted for.

Mg: milligram. µg: microgram.

**
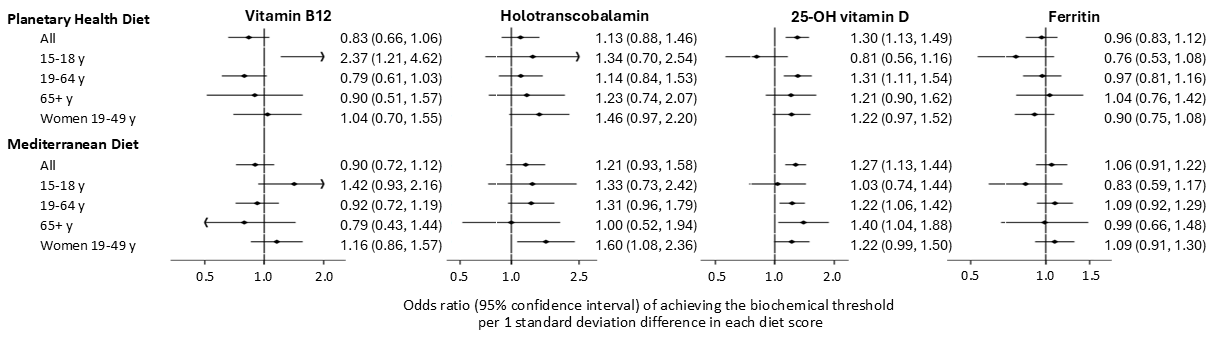
**

# Supplementary Figure 4. Comparison between adjusted odds ratios (95% Confidence Interval) of meeting the biochemical threshold with increasing standardized planetary health diet score and standardized Mediterranean diet score for selected nutrients by age group.

Vitamin B12 (pmol/L), Holotranscobalamin (pmol/L), 25-OH vitamin D (nmol/L) and Ferritin (µg/L). Logistic regression models adjusted for age, sex, ethnicity, occupation, income, region, survey year, season, body-mass index, smoking, alcohol consumption, supplement use, physical activity energy expenditure, chronic health conditions and total energy intake. Standardized diet scores were calculated by subtracting the population mean from the raw score and then dividing by the population standard deviation for each diet and each age group in turn. An odds ratio of 1 represents no association, <1 represents an inverse association and >1 represents a positive association between the diet score and the likelihood of reaching the reference nutrient intake. Models were conducted using the survey design and sampling weights accounted for.

Odds Ratio (95% Confidence Interval) of achieving the biochemical threshold with higher standardized diet scores

L: liters. nmol/nanomole. pmol: picomole. 25-OH vitamin D: 25-hydroxy vitamin D µg: microgram.

# Supplementary Table 1. Planetary health diet and Mediterranean diet scores based on scoring systems described elsewhere. ^1, 2^

PHD scoring out of a maximum of 140.

| **PHD**  **Component** | **EAT-Lancet reference diet  (for 2500 kcal/d)** | | **Criteria for scoring for PHD (range 0-140)**  **based on amount consumed (g/d)** | | |
| --- | --- | --- | --- | --- | --- |
|  | **g/day (uncertainty range)** | **kcal/day** | **Min score (0)** | **Max score (10)** | **Weight in total score** |
| Whole grains (dry weight) | 232 (0-60% of TEI) | 811 | 0 | Females: ≥ 75  Males: ≥ 90 | 1 |
| Starchy vegetables | 50 (0-100) | 39 | ≥ 200 | ≤ 50 | 1 |
| Vegetables | 300 (200-600) | 78 | 0 | ≥ 300 | 1 |
| Fruits | 200 (100-300) | 126 | 0 | ≥ 200 | 1 |
| Dairy foods | 250 (0-500) | 153 | ≥ 1000 | ≤ 250 | 1 |
| Red/processed meat | 14 (0-28) | 30 | ≥ 100 | ≤ 14 | 1 |
| Chicken and other poultry | 29 (0-58) | 62 | ≥ 100 | ≤ 29 | 1 |
| Eggs | 12 (0-25) | 19 | ≥ 120 | ≤ 12 | 1 |
| Fish | 28 (0-100) | 40 | 0 | ≥ 28 | 1 |
| Nuts | 50 (0-75) | 291 | 0 | ≥ 50 | 1 |
| Non-soy legumes | 75 (0-150) | 284 | 0 | ≥100 | 0.5 |
| Soy foods | 25 (0-50) | 112 | 0 | ≥50 | 0.5 |
| Added fat – unsaturated oils | 40 (20-80) | 354 (14.16% of TEI) | ≤ 3.5% of TEI | ≥ 21% of TEI | 1 |
| Added fat – saturated oils, trans-fat | 11.8 (0-11.8) | 96 (3.8% of TEI) | ≥ 10% of TEI | 0% of TEI | 1 |
| Added sugar and fruit juice | 31 (0-31) | 120 (4.8% of TEI) | ≥25% of TEI | <5% of TEI | 1 |

TEI: Total energy intake. Kcal: kilocalories. D: day. G: grams. PHD: planetary health diet.

Pyramid based MD scoring criteria out of a maximum of 15.

| **MD Component** | **Recommended number of servings** | **Score of 0** | **Score of 1** |
| --- | --- | --- | --- |
| Vegetables | ≥6/d | 0/d | ≥6/d |
| Legumes | ≥2/wk | 0/wk | ≥2/wk |
| Fruits | 3-6/d | 0/d | 3-6/d |
| Nuts | 1-2/d | 0/d | 1-2/d |
| Cereals | 3-6/d | 0/d | 3-6/d |
| Dairy | 2/d | 0/d | 1.5-2.5/d |
| Fish | ≥2/wk | 0/wk | ≥2/wk |
| Red meat | ˂2/wk | ≥4/wk | ˂2/wk |
| Processed meat | ≤1/wk | ≥2/wk | ≤1/wk |
| White meat | 2/wk | 0/wk | 1.5-2.5/wk |
| Egg | 2-4/wk | 0/wk | 2-4/wk |
| Potato | ≤3/wk | ≥6/wk | ≤3/wk |
| Sweets | ≤2/wk | ≥4/wk | ≤2/wk |
| Alcohol | 2/d for males,  1/d for females | ≥4/d for males,  ≥2/d for females | 1.5-2.5/d for males,  0.5-1.5/d for females |
| Olive oil | Principal source of  dietary lipids | Non-consumers | Consumers |

D: day. Wk: week. MD: Mediterranean diet.

# Supplementary Table 2. Reference Nutrient Intakes by age and sex for dietary vitamins in the United Kingdom.^3^

| **Age** | **Thiamine mg/d** | **Riboflavin mg/d** | **Niacin mg/d** | **Vitamin B6 mg/d** | **Vitamin B12 μg/d** | **Folate μg/d** | **Vitamin C mg/d** | **Vitamin A μg/d** | **Vitamin D μg/d** |
| --- | --- | --- | --- | --- | --- | --- | --- | --- | --- |
| **Males** | | | | | | | | | |
| 15-18 years | 1.1 | 1.3 | 18 | 1.5 | 1.5 | 200 | 40 | 700 | 10 |
| 19-50 years | 1 | 1.3 | 17 | 1.4 | 1.5 | 200 | 40 | 700 | 10 |
| 50+ years | 0.9 | 1.3 | 16 | 1.4 | 1.5 | 200 | 40 | 700 | 10 |
| **Females** | | | | | | | | | |
| 15-18 years | 0.8 | 1.1 | 14 | 1.2 | 1.5 | 200 | 40 | 600 | 10 |
| 19-50 years | 0.8 | 1.1 | 13 | 1.2 | 1.5 | 200 | 40 | 600 | 10 |
| 50+ years | 0.8 | 1.1 | 12 | 1.2 | 1.5 | 200 | 40 | 600 | 10 |

Mg/d: milligram per day, μg/d: microgram per day.

# Supplementary Table 3. Reference Nutrient Intakes by age and sex for dietary minerals and dietary reference value (DRV) for sodium intake in the United Kingdom.^3^.

| **Age** | **Calcium mg/d** | **Phosphorus mg/d** | **Magnesium mg/d** | **Sodium mg/d** | **Sodium DRV mg/d** | **Potassium mg/d** | **Chloride mg/d** | **Iron mg/d** | **Zinc mg/d** | **Copper mg/d** | **Selenium μg/d** | **Iodine μg/d** |
| --- | --- | --- | --- | --- | --- | --- | --- | --- | --- | --- | --- | --- |
| **Males** | | | | | | | | | | | | |
| 15-18 years | 1000 | 775 | 300 | 1600 | ≤2400 | 3500 | 2500 | 11.3 | 9.5 | 1 | 70 | 140 |
| 19-50 years | 700 | 550 | 300 | 1600 | ≤2400 | 3500 | 2500 | 8.7 | 9.5 | 1.2 | 75 | 140 |
| 50+ years | 700 | 550 | 300 | 1600 | ≤2400 | 3500 | 2500 | 8.7 | 9.5 | 1.2 | 75 | 140 |
| **Females** | | | | | | | | | | | | |
| 15-18 years | 800 | 625 | 300 | 1600 | ≤2400 | 3500 | 2500 | 14.85 | 7 | 1 | 60 | 140 |
| 19-50 years | 700 | 550 | 270 | 1600 | ≤2400 | 3500 | 2500 | 14.85 | 7 | 1.2 | 60 | 140 |
| 50+ years | 700 | 550 | 270 | 1600 | ≤2400 | 3500 | 2500 | 8.7 | 7 | 1.2 | 60 | 140 |

Mg/d: milligram per day, μg/d: microgram per day.

# Supplementary Table 4. Estimated average reference (EAR) by age and sex for Energy intake, Reference nutrient intake (RNI) for protein, and dietary reference values for fiber, total carbohydrate, free sugar, fat and saturated fat intakes per day for macronutrients in the United Kingdom.^3^

| **Age** | **Energy kcal/day (EAR)** | **Protein g/kg body weight/d (RNI)** | **Fiber g/day** | **Total carbohydrate as a % of total energy intake** | **Total free sugars as a % of total energy intake** | **Total fat as a % of total energy intake** | **Total saturated fat as a % of total energy intake** |
| --- | --- | --- | --- | --- | --- | --- | --- |
| **Males** |  |  |  |  |  |  |  |
| 15 years | 2820 | 0.75 | 25 | 50% | ≤5% | ≤35% | ≤11% |
| 16 years | 2964 | 0.75 | 25 | 50% | ≤5% | ≤35% | ≤11% |
| 17 years | 3083 | 0.75 | 30 | 50% | ≤5% | ≤35% | ≤11% |
| 18 years | 3155 | 0.75 | 30 | 50% | ≤5% | ≤35% | ≤11% |
| 19-24 years | 2772 | 0.75 | 30 | 50% | ≤5% | ≤35% | ≤11% |
| 25-34 years | 2749 | 0.75 | 30 | 50% | ≤5% | ≤35% | ≤11% |
| 35-44 years | 2629 | 0.75 | 30 | 50% | ≤5% | ≤35% | ≤11% |
| 45-54 years | 2581 | 0.75 | 30 | 50% | ≤5% | ≤35% | ≤11% |
| 55-64 years | 2581 | 0.75 | 30 | 50% | ≤5% | ≤35% | ≤11% |
| 65-74 years | 2342 | 0.75 | 30 | 50% | ≤5% | ≤35% | ≤11% |
| 75 years + | 2294 | 0.75 | 30 | 50% | ≤5% | ≤35% | ≤11% |
| **Females** |  |  |  |  |  |  |  |
| 15 years | 2390 | 0.75 | 25 | 50% | ≤5% | ≤35% | ≤11% |
| 16 years | 2414 | 0.75 | 25 | 50% | ≤5% | ≤35% | ≤11% |
| 17 years | 2462 | 0.75 | 30 | 50% | ≤5% | ≤35% | ≤11% |
| 18 years | 2462 | 0.75 | 30 | 50% | ≤5% | ≤35% | ≤11% |
| 19-24 years | 2175 | 0.75 | 30 | 50% | ≤5% | ≤35% | ≤11% |
| 25-34 years | 2175 | 0.75 | 30 | 50% | ≤5% | ≤35% | ≤11% |
| 35-44 years | 2103 | 0.75 | 30 | 50% | ≤5% | ≤35% | ≤11% |
| 45-54 years | 2103 | 0.75 | 30 | 50% | ≤5% | ≤35% | ≤11% |
| 55-64 years | 2079 | 0.75 | 30 | 50% | ≤5% | ≤35% | ≤11% |
| 65-74 years | 1912 | 0.75 | 30 | 50% | ≤5% | ≤35% | ≤11% |
| 75 years + | 1840 | 0.75 | 30 | 50% | ≤5% | ≤35% | ≤11% |

D: day. EAR: Estimated average requirement. G: grams. Kg: kilogram. RNI: Reference nutrient intake.

# Supplementary Table 5. Nutritional biomarker assays and cut-offs as used in the National Diet and Nutrition Survey in the United Kingdom.*^4, 5^

| Nutritional biomarkers | Assay information | %CV* | Limit of detection | Sex-specific cutoffs for adequacy for individuals aged >15 years | |
| --- | --- | --- | --- | --- | --- |
|  |  |  |  | Males | Females |
| Ferritin, µg/L | Immunonephelometry, Siemens BN ProSpec | 3.1-10.1 | 0.00078‡ | ≥15 | ≥15 |
| Vitamin B12, pmol/L | Competitive immunoassay, ADVIA Centaur B12 Assay | 3.6-19.0 | 33‡ | ≥150 | ≥150 |
| HoloTC, pmol/L | Enzyme-linked immunosorbent assay, Axis Shield | 8.7-11.6 | 8.1‡ | ≥32 | ≥32 |
| Riboflavin, (unit less) | Erythrocyte glutathione reductase activation coefficient, Cobas Fara centrifugal analyser. | 1.1-7.3 | Not available | <1.3 | <1.3 |
| Folate, nmol/L | Liquid chromatography-tandem mass spectrometry, Waters ACQUITY UPLC | 4.0-8.6 | ~1.0§ | ≥13 | ≥13 |
| 25-OHD, nmol/L | Chemiluminescent immunoassay or liquid chromatography-tandem mass spectrometry, Waters ACQUITY UPLC | 2.8-10.9 | 2.0‡ | ≥25 | ≥25 |
| Vitamin C, µmol/L | Quinoxaline assay, BMG Labtech FLUOstar OPTIMA plate reader | 3.4-13.4 | 1.1‡ | ≥11 | ≥11 |
| Urinary iodine, µg/L | ^127^I assay, inductively coupled plasma mass spectrometer | 2.0-3.0 | 23-30‡ | ≥ 100 | ≥ 100 |
| * The assay information has been available in the appendix of the survey documents issued by the survey cycles. † Range of %CV from the NDNS internal quality checks  ‡ from product, patent or literature information: ferritin,^6^ serum vitamin B12,^7^ holotranscobalamin (HoloTC),^8^ 25-hydroxy vitamin D (25-OHD),^9^ vitamin C,^10^ and for iodine.^11^  § Serum folate concentrations were the sum of six folate derivatives. Of those, folic acid and 5-methyltetrahydrofolate gave limits of detection as 0.21 and 0.26 nmol/L.^12^ Assuming the other four folate derivatives to have 0.26 nmol/L of LOD, LOD_sum_ was calculated by obtaining root-sum-squares as 0.62 nmol/L.  CV: Coefficient of variation. HoloTC: Holotranscobalamin. LOD: Limit of detection. NDNS: National Diet and Nutrition Survey. Nmol/L: nanomole per liter. Pmol/L: picomole per liter. µg/L: microgram per liter. µmol/L: micromole per liter. 25-OHD: 25-hydroxy vitamin D. | | | | | |

# Supplementary Table 6. Nutrient intake per day in participants ≥15 years in the UK National Diet and Nutrition Survey 2008-2019 (n=9671).

| **Nutrient intake per day** | **Total Population  (n=9671)** | **PHD Quintile 1***  **(n=1935)** | **PHD Quintile 2  (n=1934)** | **PHD Quintile 3  (n=1934)** | **PHD Quintile 4  (n=1934)** | **PHD Quintile 5 (n=1934)** | **P trend†** |
| --- | --- | --- | --- | --- | --- | --- | --- |
| **Energy, kcal/day*** | 1,719 (1,704, 1,735) | 1,863 (1,824, 1,904) | 1,716 (1,682, 1,750) | 1,691 (1,658, 1,725) | 1,678 (1,648, 1,709) | 1,684 (1,655, 1,714) | <0.001 |
| EAR%‡ | 13.4 | 16.5 | 12.2 | 11.9 | 12.7 | 14.0 |  |
| Crude OR (95% CI) | 0.94 (0.82, 1.07)¶ | 1.00 (Ref) | 0.70 (0.54, 0.91) | 0.68 (0.52, 0.88) | 0.74 (0.58, 0.95) | 0.82 (0.64, 1.06) | 0.339 |
| Adjusted OR§ | 0.90 (0.76, 1.07)¶ | 1.00 (Ref) | 0.69 (0.52, 0.91) | 0.61 (0.45, 0.83) | 0.68 (0.51, 0.89) | 0.75 (0.56, 1.02) | 0.187 |
|  |  |  |  |  |  |  |  |
| **Protein, g/Kg body weight*** | 0.9 (0.9, 0.9) | 0.9 (0.9, 1.0) | 0.9 (0.9, 0.9) | 0.9 (0.9, 0.9) | 0.9 (0.9, 1.0) | 1.0 (0.9, 1.0) | 0.092 |
| RNI%‡ | 75.7 | 75.6 | 73.8 | 73.1 | 76.9 | 78.6 |  |
| Crude OR (95% CI) | 1.09 (1.00, 1.20)¶ | 1.00 (Ref) | 0.91 (0.74, 1.13) | 0.87 (0.71, 1.08) | 1.07 (0.87, 1.32) | 1.18 (0.95, 1.47) | 0.025 |
| Adjusted OR§ | 0.87 (0.77, 0.98)¶ | 1.00 (Ref) | 0.93 (0.72, 1.19) | 0.81 (0.64, 1.04) | 0.92 (0.72, 1.18) | 0.79 (0.61, 1.03) | 0.111 |
| + energy-adjusted | 0.98 (0.85, 1.14)¶ | 1.00 (Ref) | 1.19 (0.90, 1.59) | 1.13 (0.84, 1.51) | 1.30 (0.97, 1.74) | 1.04 (0.77, 1.41) | 0.746 |
|  |  |  |  |  |  |  |  |
| **Fiber, g*** | 17.2 (17.0, 17.4) | 13.9 (13.6, 14.3) | 14.8 (14.5, 15.1) | 16.2 (15.9, 16.6) | 18.3 (18.0, 18.6) | 22.4 (22.0, 22.8) | <0.001 |
| DRV%‡ | 6.4 | 1.5 | 1.9 | 3.0 | 5.3 | 17.3 |  |
| Crude OR (95% CI) | 4.88 (3.95, 6.03)¶ | 1.00 (Ref) | 1.27 (0.60, 2.68) | 2.00 (0.98, 4.07) | 3.63 (1.91, 6.89) | 13.49 (7.35, 24.72) | <0.001 |
| Adjusted OR§ | 5.88 (4.63, 7.47)¶ | 1.00 (Ref) | 1.69 (0.77, 3.68) | 2.66 (1.25, 5.65) | 5.40 (2.70, 10.82) | 20.51 (10.53, 39.93) | <0.001 |
| + energy-adjusted | 9.93 (7.70, 12.81)¶ | 1.00 (Ref) | 3.71 (1.64, 8.43) | 5.93 (2.64, 13.34) | 17.67 (8.38, 37.27) | 87.78 (41.40, 186.08) | <0.001 |
|  |  |  |  |  |  |  |  |
| **Carbohydrate,** % energy* | 45.2 (45.0, 45.4) | 45.2 (44.7, 45.7) | 44.8 (44.3, 45.3) | 45.0 (44.5, 45.5) | 45.3 (44.8, 45.7) | 45.7 (45.2, 46.2) | 0.041 |
| DRV%‡ | 28.7 | 27.7 | 25.9 | 27.4 | 29.8 | 31.6 |  |
| Crude OR (95% CI) | 1.15 (1.05, 1.26)¶ | 1.00 (Ref) | 0.91 (0.75, 1.12) | 0.99 (0.81, 1.20) | 1.11 (0.91, 1.35) | 1.20 (0.99, 1.46) | 0.008 |
| Adjusted OR§ | 1.33 (1.19, 1.49)¶ | 1.00 (Ref) | 0.95 (0.75, 1.20) | 1.16 (0.91, 1.47) | 1.44 (1.13, 1.82) | 1.52 (1.19, 1.96) | <0.001 |
| + energy-adjusted | 1.32 (1.17, 1.48)¶ | 1.00 (Ref) | 0.93 (0.74, 1.17) | 1.13 (0.89, 1.43) | 1.40 (1.10, 1.77) | 1.49 (1.16, 1.91) | <0.001 |
|  |  |  |  |  |  |  |  |
| **Free sugars, %energy*** | 9.7 (9.5, 9.8) | 13.6 (13.1, 14.1) | 10.8 (10.4, 11.2) | 9.9 (9.6, 10.2) | 9.0 (8.8, 9.3) | 7.2 (6.9, 7.5) | <0.001 |
| DRV%‡ | 12.6 | 6.0 | 9.5 | 10.2 | 13.4 | 21.0 |  |
| Crude OR (95% CI) | 2.10 (1.85, 2.38)¶ | 1.00 (Ref) | 1.63 (1.16, 2.29) | 1.77 (1.28, 2.46) | 2.41 (1.75, 3.32) | 4.16 (3.07, 5.65) | <0.001 |
| Adjusted OR§ | 2.31 (1.98, 2.70)¶ | 1.00 (Ref) | 1.62 (1.12, 2.34) | 1.72 (1.20, 2.45) | 2.34 (1.64, 3.34) | 4.54 (3.21, 6.42) | <0.001 |
| + energy-adjusted | 2.34 (1.98, 2.76)¶ | 1.00 (Ref) | 1.52 (1.05, 2.20) | 1.54 (1.07, 2.21) | 2.14 (1.49, 3.07) | 4.26 (2.98, 6.08) | <0.001 |
|  |  |  |  |  |  |  |  |
| **Total fat, %energy*** | 32.7 (32.5, 32.9) | 33.4 (33.0, 33.8) | 33.2 (32.8, 33.6) | 32.7 (32.3, 33.2) | 32.4 (32.0, 32.8) | 32.2 (31.8, 32.7) | <0.001 |
| DRV%‡ | 60.3 | 58.7 | 57.6 | 59.5 | 63.8 | 61.2 |  |
| Crude OR (95% CI) | 1.08 (0.99, 1.17)¶ | 1.00 (Ref) | 0.96 (0.80, 1.14) | 1.04 (0.87, 1.24) | 1.24 (1.04, 1.48) | 1.11 (0.93, 1.33) | 0.025 |
| Adjusted OR§ | 1.21 (1.09, 1.34)¶ | 1.00 (Ref) | 1.01 (0.83, 1.22) | 1.10 (0.91, 1.35) | 1.47 (1.20, 1.80) | 1.38 (1.12, 1.70) | <0.001 |
| + energy-adjusted | 1.17 (1.06, 1.30)¶ | 1.00 (Ref) | 0.93 (0.77, 1.13) | 1.01 (0.82, 1.24) | 1.36 (1.11, 1.68) | 1.28 (1.03, 1.59) | <0.001 |
|  |  |  |  |  |  |  |  |
| **Saturated fat, %energy*** | 11.8 (11.8, 11.9) | 12.3 (12.1, 12.5) | 12.4 (12.2, 12.6) | 12.1 (11.9, 12.3) | 11.8 (11.6, 12.0) | 10.9 (10.7, 11.2) | <0.001 |
| DRV%‡ | 35.6 | 30.1 | 28.9 | 31.6 | 36.9 | 46.9 |  |
| Crude OR (95% CI) | 1.53 (1.40, 1.66)¶ | 1.00 (Ref) | 0.94 (0.77, 1.15) | 1.07 (0.88, 1.30) | 1.36 (1.11, 1.65) | 2.05 (1.70, 2.46) | <0.001 |
| Adjusted OR§ | 1.88 (1.69, 2.08)¶ | 1.00 (Ref) | 1.00 (0.81, 1.23) | 1.23 (0.99, 1.52) | 1.73 (1.39, 2.17) | 2.97 (2.39, 3.70) | <0.001 |
| + energy-adjusted | 1.89 (1.70, 2.11)¶ | 1.00 (Ref) | 0.91 (0.73, 1.14) | 1.11 (0.89, 1.38) | 1.63 (1.29, 2.05) | 2.90 (2.31, 3.63) | <0.001 |
|  |  |  |  |  |  |  |  |
| **Thiamine, mg*** | 1.4 (1.4, 1.4) | 1.3 (1.3, 1.4) | 1.3 (1.3, 1.4) | 1.3 (1.3, 1.4) | 1.4 (1.4, 1.4) | 1.4 (1.4, 1.5) | <0.001 |
| RNI%‡ | 89.5 | 84.2 | 86.4 | 87.8 | 92.0 | 94.8 |  |
| Crude OR (95% CI) | 1.81 (1.60, 2.05)¶ | 1.00 (Ref) | 1.20 (0.93, 1.54) | 1.36 (1.04, 1.77) | 2.16 (1.64, 2.84) | 3.46 (2.49, 4.80) | <0.001 |
| Adjusted OR§ | 1.50 (1.30, 1.74)¶ | 1.00 (Ref) | 1.10 (0.83, 1.46) | 1.06 (0.80, 1.42) | 1.69 (1.25, 2.30) | 2.34 (1.60, 3.43) | <0.001 |
| + energy-adjusted | 2.37 (1.96, 2.87)¶ | 1.00 (Ref) | 1.56 (1.10, 2.22) | 1.77 (1.23, 2.55) | 3.02 (2.12, 4.32) | 4.70 (3.11, 7.10) | <0.001 |
|  |  |  |  |  |  |  |  |
| **Riboflavin, mg*** | 1.4 (1.4, 1.5) | 1.4 (1.4, 1.4) | 1.4 (1.4, 1.5) | 1.4 (1.4, 1.5) | 1.5 (1.5, 1.5) | 1.5 (1.4, 1.5) | <0.001 |
| RNI%‡ | 70.0 | 62.4 | 67.9 | 68.1 | 75.3 | 74.1 |  |
| Crude OR (95% CI) | 1.32 (1.20, 1.44)¶ | 1.00 (Ref) | 1.28 (1.05, 1.54) | 1.29 (1.07, 1.55) | 1.84 (1.53, 2.22) | 1.73 (1.42, 2.10) | <0.001 |
| Adjusted OR§ | 1.18 (1.06, 1.32)¶ | 1.00 (Ref) | 1.19 (0.96, 1.47) | 1.11 (0.90, 1.38) | 1.57 (1.26, 1.95) | 1.41 (1.11, 1.78) | <0.001 |
| + energy-adjusted | 1.42 (1.25, 1.62)¶ | 1.00 (Ref) | 1.54 (1.21, 1.96) | 1.53 (1.19, 1.97) | 2.28 (1.78, 2.91) | 2.04 (1.56, 2.67) | <0.001 |
|  |  |  |  |  |  |  |  |
| **Niacin, mg*** | 33.0 (32.7, 33.3) | 35.4 (34.4, 36.3) | 32.9 (32.1, 33.6) | 32.4 (31.6, 33.1) | 32.7 (32.0, 33.4) | 32.3 (31.7, 32.9) | <0.001 |
| RNI%‡ | 98.5 | 98.6 | 97.7 | 98.0 | 98.2 | 99.7 |  |
| Crude OR (95% CI) | 1.56 (1.29, 1.88)¶ | 1.00 (Ref) | 0.59 (0.34, 1.03) | 0.71 (0.38, 1.31) | 0.79 (0.42, 1.50) | 4.24 (1.54, 11.68) | <0.001 |
| Adjusted OR§ | 1.26 (1.01, 1.57)¶ | 1.00 (Ref) | 0.49 (0.26, 0.91) | 0.54 (0.28, 1.06) | 0.52 (0.26, 1.04) | 2.29 (0.83, 6.33) | 0.196 |
| + energy-adjusted | 2.28 (1.54, 3.39)¶ | 1.00 (Ref) | 0.82 (0.38, 1.81) | 1.09 (0.46, 2.59) | 1.03 (0.44, 2.40) | 4.68 (1.44, 15.21) | 0.004 |
|  |  |  |  |  |  |  |  |
| **Vitamin B6, mg*** | 1.8 (1.8, 1.8) | 1.9 (1.9, 2.0) | 1.8 (1.7, 1.8) | 1.7 (1.7, 1.8) | 1.8 (1.7, 1.8) | 1.8 (1.8, 1.8) | 0.020 |
| RNI%‡ | 79.9 | 78.7 | 77.4 | 76.7 | 81.8 | 83.9 |  |
| Crude OR (95% CI) | 1.21 (1.11, 1.33)¶ | 1.00 (Ref) | 0.93 (0.74, 1.15) | 0.89 (0.72, 1.11) | 1.22 (0.98, 1.50) | 1.41 (1.13, 1.76) | <0.001 |
| Adjusted OR§ | 1.19 (1.06, 1.34)¶ | 1.00 (Ref) | 0.97 (0.76, 1.23) | 0.87 (0.68, 1.10) | 1.18 (0.92, 1.50) | 1.36 (1.04, 1.79) | 0.004 |
| + energy-adjusted | 1.41 (1.22, 1.62)¶ | 1.00 (Ref) | 1.16 (0.88, 1.54) | 1.08 (0.81, 1.44) | 1.53 (1.17, 2.01) | 1.85 (1.37, 2.51) | <0.001 |
|  |  |  |  |  |  |  |  |
| **Vitamin B12, ug*** | 4.4 (4.4, 4.5) | 4.3 (4.2, 4.5) | 4.4 (4.2, 4.5) | 4.3 (4.1, 4.4) | 4.6 (4.5, 4.8) | 4.6 (4.4, 4.7) | 0.004 |
| RNI%‡ | 96.2 | 97.0 | 96.5 | 95.5 | 95.9 | 96.1 |  |
| Crude OR (95% CI) | 0.83 (0.67, 1.03)¶ | 1.00 (Ref) | 0.86 (0.53, 1.37) | 0.66 (0.42, 1.04) | 0.72 (0.44, 1.17) | 0.75 (0.47, 1.20) | 0.203 |
| Adjusted OR§ | 0.72 (0.57, 0.92)¶ | 1.00 (Ref) | 0.70 (0.42, 1.18) | 0.50 (0.30, 0.82) | 0.52 (0.30, 0.90) | 0.54 (0.32, 0.92) | 0.013 |
| + energy-adjusted | 0.80 (0.59, 1.08)¶ | 1.00 (Ref) | 0.86 (0.50, 1.47) | 0.65 (0.38, 1.11) | 0.68 (0.38, 1.20) | 0.73 (0.41, 1.31) | 0.242 |
|  |  |  |  |  |  |  |  |
| **Folate, µg*** | 225 (223, 228) | 201 (195, 207) | 205 (199, 210) | 215 (210, 221) | 238 (233, 244) | 262 (256, 267) | <0.001 |
| RNI%‡ | 63.6 | 50.8 | 53.4 | 60.5 | 68.8 | 78.7 |  |
| Crude OR (95% CI) | 1.95 (1.80, 2.13)¶ | 1.00 (Ref) | 1.11 (0.93, 1.33) | 1.48 (1.24, 1.77) | 2.14 (1.79, 2.55) | 3.58 (2.97, 4.31) | <0.001 |
| Adjusted OR§ | 2.19 (1.96, 2.46)¶ | 1.00 (Ref) | 1.20 (0.97, 1.48) | 1.66 (1.34, 2.05) | 2.55 (2.05, 3.18) | 4.25 (3.35, 5.38) | <0.001 |
| + energy-adjusted | 3.03 (2.65, 3.46)¶ | 1.00 (Ref) | 1.56 (1.22, 2.01) | 2.47 (1.91, 3.19) | 4.04 (3.12, 5.25) | 7.71 (5.86, 10.15) | <0.001 |
|  |  |  |  |  |  |  |  |
| **Vitamin C, mg*** | 65.4 (64.1, 66.6) | 49.7 (47.3, 52.2) | 52.3 (49.9, 54.8) | 60.3 (57.9, 62.8) | 72.4 (69.9, 75.0) | 92.8 (90.0, 95.7) | <0.001 |
| RNI%‡ | 77.3 | 63.6 | 66.0 | 74.8 | 83.5 | 92.8 |  |
| Crude OR (95% CI) | 2.66 (2.41, 2.94)¶ | 1.00 (Ref) | 1.11 (0.92, 1.33) | 1.70 (1.40, 2.05) | 2.89 (2.37, 3.52) | 7.40 (5.75, 9.52) | <0.001 |
| Adjusted OR§ | 2.39 (2.11, 2.70)¶ | 1.00 (Ref) | 1.05 (0.85, 1.29) | 1.54 (1.25, 1.89) | 2.57 (2.03, 3.25) | 5.57 (4.18, 7.42) | <0.001 |
| + energy-adjusted | 2.85 (2.49, 3.25)¶ | 1.00 (Ref) | 1.19 (0.96, 1.48) | 1.87 (1.50, 2.32) | 3.16 (2.47, 4.03) | 7.03 (5.25, 9.41) | <0.001 |
|  |  |  |  |  |  |  |  |
| **Vitamin A, µg of RE*** | 706 (691, 721) | 573 (544, 603) | 626 (600, 653) | 674 (645, 703) | 762 (730, 796) | 877 (844, 912) | <0.001 |
| RNI%‡ | 55.3 | 42.5 | 47.6 | 52.7 | 60.7 | 68.1 |  |
| Crude OR (95% CI) | 1.78 (1.64, 1.93)¶ | 1.00 (Ref) | 1.23 (1.03, 1.47) | 1.51 (1.27, 1.79) | 2.08 (1.75, 2.48) | 2.89 (2.43, 3.43) | <0.001 |
| Adjusted OR§ | 1.51 (1.37, 1.67)¶ | 1.00 (Ref) | 1.09 (0.90, 1.33) | 1.23 (1.01, 1.48) | 1.57 (1.28, 1.92) | 2.03 (1.65, 2.49) | <0.001 |
| + energy-adjusted | 1.68 (1.50, 1.87)¶ | 1.00 (Ref) | 1.25 (1.01, 1.54) | 1.45 (1.18, 1.79) | 1.87 (1.51, 2.32) | 2.48 (1.98, 3.11) | <0.001 |
|  |  |  |  |  |  |  |  |
| **Vitamin D, µg*** | 2.2 (2.2, 2.3) | 2.1 (2.0, 2.2) | 2.1 (2.0, 2.2) | 2.1 (2.0, 2.2) | 2.3 (2.2, 2.4) | 2.6 (2.5, 2.7) | <0.001 |
| RNI%‡ | 1.3 | 0.0 | 0.8 | 1.0 | 1.4 | 2.6 |  |
| Crude OR (95% CI) | 2.95 (2.07, 4.20)¶ | 1.00 (Ref) | 17.68 (4.28, 73.14) | 23.43 (6.07, 90.41) | 31.82 (8.72, 116.12) | 58.76 (16.67, 207.12) | <0.001 |
| Adjusted OR§ | 3.15 (2.04, 4.84)¶ | 1.00 (Ref) | 23.11 (4.45, 120.06) | 31.26 (6.50, 150.38) | 43.00 (9.72, 190.19) | 71.62 (15.73, 326.09) | <0.001 |
| + energy-adjusted | 3.35 (2.17, 5.16)¶ | 1.00 (Ref) | 39.75 (6.19, 255.06) | 49.77 (8.49, 291.74) | 74.95 (13.15, 427.26) | 125.57 (21.69, 726.84) | <0.001 |
|  |  |  |  |  |  |  |  |
| **Calcium, mg*** | 751 (743, 760) | 714 (694, 735) | 740 (722, 759) | 742 (724, 760) | 763 (746, 781) | 787 (769, 804) | <0.001 |
| RNI%‡ | 58.5 | 51.5 | 56.1 | 56.6 | 61.6 | 64.4 |  |
| Crude OR (95% CI) | 1.31 (1.21, 1.42)¶ | 1.00 (Ref) | 1.20 (1.01, 1.44) | 1.23 (1.03, 1.46) | 1.51 (1.27, 1.80) | 1.71 (1.43, 2.03) | <0.001 |
| Adjusted OR§ | 1.35 (1.23, 1.49)¶ | 1.00 (Ref) | 1.22 (0.99, 1.49) | 1.22 (1.00, 1.50) | 1.59 (1.30, 1.95) | 1.75 (1.42, 2.15) | <0.001 |
| + energy-adjusted | 1.81 (1.60, 2.04)¶ | 1.00 (Ref) | 1.73 (1.36, 2.19) | 1.92 (1.50, 2.45) | 2.60 (2.05, 3.31) | 3.15 (2.45, 4.06) | <0.001 |
|  |  |  |  |  |  |  |  |
| **Phosphorus, mg*** | 1,160 (1,150, 1,171) | 1,149 (1,123, 1,176) | 1,119 (1,097, 1,142) | 1,128 (1,104, 1,151) | 1,169 (1,148, 1,191) | 1,222 (1,201, 1,244) | <0.001 |
| RNI%‡ | 97.4 | 96.7 | 96.4 | 97.0 | 97.6 | 98.8 |  |
| Crude OR (95% CI) | 1.55 (1.31, 1.83)¶ | 1.00 (Ref) | 0.91 (0.59, 1.39) | 1.09 (0.70, 1.68) | 1.40 (0.87, 2.26) | 2.77 (1.57, 4.88) | <0.001 |
| Adjusted OR§ | 1.31 (1.05, 1.64)¶ | 1.00 (Ref) | 0.85 (0.53, 1.38) | 0.85 (0.51, 1.40) | 1.05 (0.61, 1.81) | 2.05 (1.04, 4.05) | 0.032 |
| + energy-adjusted | 2.83 (1.92, 4.17)¶ | 1.00 (Ref) | 1.60 (0.82, 3.12) | 2.16 (1.13, 4.13) | 3.34 (1.65, 6.74) | 5.62 (2.48, 12.75) | <0.001 |
|  |  |  |  |  |  |  |  |
| **Magnesium, mg*** | 241 (239, 244) | 214 (209, 219) | 216 (211, 221) | 230 (225, 235) | 251 (246, 255) | 289 (284, 294) | <0.001 |
| RNI%‡ | 32.1 | 16.7 | 20.1 | 25.1 | 37.0 | 54.1 |  |
| Crude OR (95% CI) | 2.76 (2.51, 3.05)¶ | 1.00 (Ref) | 1.26 (0.99, 1.60) | 1.67 (1.33, 2.10) | 2.93 (2.38, 3.62) | 5.91 (4.79, 7.27) | <0.001 |
| Adjusted OR§ | 3.32 (2.94, 3.75)¶ | 1.00 (Ref) | 1.44 (1.10, 1.88) | 2.03 (1.56, 2.63) | 3.99 (3.09, 5.15) | 8.19 (6.34, 10.59) | <0.001 |
| + energy-adjusted | 8.59 (7.20, 10.24)¶ | 1.00 (Ref) | 2.94 (2.02, 4.27) | 5.56 (3.82, 8.09) | 15.61 (10.78, 22.62) | 51.98 (35.45, 76.20) | <0.001 |
|  |  |  |  |  |  |  |  |
| **Sodium, mg*** | 1,972 (1,950, 1,994) | 2,182 (2,124, 2,242) | 2,045 (1,997, 2,095) | 1,958 (1,912, 2,006) | 1,947 (1,902, 1,993) | 1,810 (1,769, 1,852) | <0.001 |
| RNI%‡ | 73.2 | 80.4 | 76.6 | 71.8 | 73.8 | 65.8 |  |
| Crude OR (95% CI) | 0.70 (0.64, 0.76)¶ | 1.00 (Ref) | 0.80 (0.65, 0.98) | 0.62 (0.51, 0.76) | 0.69 (0.56, 0.84) | 0.47 (0.38, 0.57) | <0.001 |
| Adjusted OR§ | 0.82 (0.73, 0.91)¶ | 1.00 (Ref) | 0.95 (0.74, 1.21) | 0.78 (0.62, 0.99) | 0.93 (0.73, 1.19) | 0.65 (0.51, 0.82) | 0.001 |
| + energy-adjusted | 0.85 (0.74, 0.98)¶ | 1.00 (Ref) | 1.19 (0.89, 1.61) | 1.04 (0.77, 1.39) | 1.19 (0.88, 1.61) | 0.78 (0.58, 1.05) | 0.051 |
|  |  |  |  |  |  |  |  |
| **Sodium, mg*** | 1,972 (1,950, 1,994) | 2,182 (2,124, 2,242) | 2,045 (1,997, 2,095) | 1,958 (1,912, 2,006) | 1,947 (1,902, 1,993) | 1,810 (1,769, 1,852) | <0.001 |
| DRV%‡ | 68.0 | 56.0 | 65.4 | 68.5 | 69.9 | 76.8 |  |
| Crude OR (95% CI) | 1.62 (1.49, 1.78)¶ | 1.00 (Ref) | 1.49 (1.24, 1.79) | 1.71 (1.42, 2.06) | 1.82 (1.51, 2.20) | 2.61 (2.15, 3.15) | <0.001 |
| Adjusted OR§ | 1.22 (1.09, 1.36)¶ | 1.00 (Ref) | 1.16 (0.93, 1.44) | 1.21 (0.98, 1.51) | 1.15 (0.91, 1.44) | 1.47 (1.16, 1.86) | 0.005 |
| + energy-adjusted | 1.15 (1.01, 1.32)¶ | 1.00 (Ref) | 1.00 (0.78, 1.29) | 1.00 (0.77, 1.32) | 0.94 (0.71, 1.23) | 1.32 (0.99, 1.76) | 0.124 |
|  |  |  |  |  |  |  |  |
| **Potassium, mg*** | 2,642 (2,618, 2,667) | 2,516 (2,460, 2,573) | 2,443 (2,392, 2,495) | 2,540 (2,488, 2,593) | 2,696 (2,648, 2,745) | 2,956 (2,909, 3,003) | <0.001 |
| RNI%‡ | 18.6 | 15.1 | 14.2 | 16.0 | 19.1 | 26.5 |  |
| Crude OR (95% CI) | 1.49 (1.33, 1.66)¶ | 1.00 (Ref) | 0.92 (0.70, 1.21) | 1.07 (0.83, 1.37) | 1.33 (1.05, 1.68) | 2.02 (1.60, 2.54) | <0.001 |
| Adjusted OR§ | 1.76 (1.54, 2.01)¶ | 1.00 (Ref) | 1.05 (0.78, 1.42) | 1.19 (0.90, 1.58) | 1.71 (1.29, 2.27) | 2.77 (2.09, 3.67) | <0.001 |
| + energy-adjusted | 2.33 (1.99, 2.72)¶ | 1.00 (Ref) | 1.40 (0.96, 2.04) | 1.72 (1.20, 2.45) | 2.80 (1.96, 4.01) | 5.23 (3.67, 7.45) | <0.001 |
|  |  |  |  |  |  |  |  |
| **Chloride, mg*** | 3,182 (3,149, 3,215) | 3,399 (3,311, 3,490) | 3,234 (3,160, 3,309) | 3,144 (3,074, 3,216) | 3,174 (3,106, 3,245) | 3,030 (2,967, 3,094) | <0.001 |
| RNI%‡ | 76.4 | 80.9 | 77.5 | 74.8 | 76.7 | 73.3 |  |
| Crude OR (95% CI) | 0.82 (0.75, 0.90)¶ | 1.00 (Ref) | 0.81 (0.66, 1.00) | 0.70 (0.57, 0.86) | 0.78 (0.63, 0.96) | 0.65 (0.53, 0.80) | <0.001 |
| Adjusted OR§ | 0.92 (0.82, 1.03)¶ | 1.00 (Ref) | 0.93 (0.73, 1.19) | 0.83 (0.66, 1.05) | 0.99 (0.77, 1.27) | 0.82 (0.64, 1.05) | 0.242 |
| + energy-adjusted | 1.06 (0.91, 1.23)¶ | 1.00 (Ref) | 1.20 (0.88, 1.62) | 1.17 (0.87, 1.58) | 1.35 (0.98, 1.85) | 1.17 (0.86, 1.60) | 0.340 |
|  |  |  |  |  |  |  |  |
| **Iron, mg*** | 9.7 (9.6, 9.8) | 8.9 (8.7, 9.1) | 9.0 (8.8, 9.2) | 9.4 (9.2, 9.6) | 10.1 (9.9, 10.3) | 10.9 (10.7, 11.1) | <0.001 |
| RNI%‡ | 49.9 | 41.4 | 42.7 | 48.4 | 53.4 | 59.7 |  |
| Crude OR (95% CI) | 1.50 (1.39, 1.63)¶ | 1.00 (Ref) | 1.06 (0.88, 1.27) | 1.33 (1.11, 1.58) | 1.62 (1.36, 1.94) | 2.10 (1.76, 2.50) | <0.001 |
| Adjusted OR§ | 1.83 (1.63, 2.04)¶ | 1.00 (Ref) | 1.15 (0.90, 1.47) | 1.44 (1.13, 1.82) | 2.15 (1.68, 2.74) | 2.85 (2.23, 3.64) | <0.001 |
| + energy-adjusted | 2.40 (2.10, 2.76)¶ | 1.00 (Ref) | 1.49 (1.13, 1.96) | 2.06 (1.58, 2.69) | 3.30 (2.46, 4.41) | 4.88 (3.64, 6.55) | <0.001 |
|  |  |  |  |  |  |  |  |
| **Zinc, mg*** | 7.9 (7.8, 8.0) | 7.9 (7.7, 8.1) | 7.9 (7.7, 8.0) | 7.8 (7.6, 7.9) | 7.9 (7.7, 8.1) | 8.1 (7.9, 8.2) | 0.158 |
| RNI%‡ | 49.9 | 45.1 | 50.4 | 45.6 | 51.4 | 55.4 |  |
| Crude OR (95% CI) | 1.22 (1.13, 1.33)¶ | 1.00 (Ref) | 1.24 (1.03, 1.48) | 1.02 (0.85, 1.22) | 1.29 (1.08, 1.53) | 1.51 (1.27, 1.81) | <0.001 |
| Adjusted OR§ | 1.08 (0.98, 1.20)¶ | 1.00 (Ref) | 1.17 (0.96, 1.43) | 0.93 (0.76, 1.14) | 1.05 (0.86, 1.29) | 1.18 (0.96, 1.46) | 0.289 |
| + energy-adjusted | 1.26 (1.13, 1.42)¶ | 1.00 (Ref) | 1.57 (1.24, 2.00) | 1.23 (0.96, 1.58) | 1.40 (1.09, 1.79) | 1.68 (1.30, 2.17) | 0.002 |
|  |  |  |  |  |  |  |  |
| **Copper, mg*** | 1.1 (1.1, 1.1) | 0.9 (0.9, 1.0) | 1.0 (0.9, 1.0) | 1.0 (1.0, 1.0) | 1.1 (1.1, 1.1) | 1.3 (1.2, 1.3) | <0.001 |
| RNI%‡ | 38.3 | 26.7 | 28.4 | 33.2 | 42.1 | 55.3 |  |
| Crude OR (95% CI) | 2.01 (1.84, 2.19)¶ | 1.00 (Ref) | 1.09 (0.89, 1.33) | 1.37 (1.13, 1.66) | 1.99 (1.65, 2.41) | 3.39 (2.81, 4.10) | <0.001 |
| Adjusted OR§ | 2.33 (2.10, 2.59)¶ | 1.00 (Ref) | 1.23 (0.98, 1.54) | 1.57 (1.26, 1.95) | 2.64 (2.12, 3.30) | 4.42 (3.51, 5.57) | <0.001 |
| + energy-adjusted | 3.93 (3.45, 4.48)¶ | 1.00 (Ref) | 1.80 (1.34, 2.42) | 2.75 (2.05, 3.68) | 5.52 (4.13, 7.38) | 12.13 (8.95, 16.43) | <0.001 |
|  |  |  |  |  |  |  |  |
| **Selenium, µg*** | 43.8 (43.3, 44.3) | 40.0 (38.9, 41.0) | 40.2 (39.3, 41.2) | 42.0 (40.9, 43.1) | 45.7 (44.6, 46.8) | 49.8 (48.6, 51.0) | <0.001 |
| RNI%‡ | 13.5 | 7.1 | 8.1 | 10.9 | 15.5 | 22.6 |  |
| Crude OR (95% CI) | 2.01 (1.76, 2.30)¶ | 1.00 (Ref) | 1.16 (0.81, 1.64) | 1.59 (1.13, 2.24) | 2.40 (1.75, 3.30) | 3.81 (2.79, 5.21) | <0.001 |
| Adjusted OR§ | 2.00 (1.72, 2.32)¶ | 1.00 (Ref) | 1.33 (0.91, 1.93) | 1.80 (1.24, 2.59) | 2.78 (1.94, 3.98) | 4.13 (2.89, 5.90) | <0.001 |
| + energy-adjusted | 2.23 (1.91, 2.60)¶ | 1.00 (Ref) | 1.71 (1.15, 2.53) | 2.42 (1.64, 3.57) | 3.89 (2.66, 5.71) | 5.95 (4.07, 8.70) | <0.001 |
|  |  |  |  |  |  |  |  |
| **Iodine, µg*** | 141.2 (139.2, 143.3) | 124.9 (120.6, 129.3) | 133.5 (129.4, 137.8) | 140.7 (136.4, 145.1) | 149.8 (145.6, 154.2) | 153.6 (149.6, 157.7) | <0.001 |
| RNI%‡ | 53.1 | 42.8 | 50.4 | 51.8 | 57.3 | 59.9 |  |
| Crude OR (95% CI) | 1.38 (1.27, 1.49)¶ | 1.00 (Ref) | 1.35 (1.13, 1.63) | 1.43 (1.19, 1.72) | 1.79 (1.51, 2.13) | 1.99 (1.67, 2.37) | <0.001 |
| Adjusted OR§ | 1.37 (1.24, 1.51)¶ | 1.00 (Ref) | 1.45 (1.17, 1.79) | 1.37 (1.11, 1.70) | 1.80 (1.46, 2.22) | 2.01 (1.62, 2.49) | <0.001 |
| + energy-adjusted | 1.59 (1.42, 1.77)¶ | 1.00 (Ref) | 1.87 (1.49, 2.35) | 1.81 (1.41, 2.31) | 2.45 (1.94, 3.09) | 2.85 (2.24, 3.63) | <0.001 |

* Geometric mean and 95% confidence intervals are reported. The score ranges of the five categories were <62.0 points for Q1, 62.0-69.8 points for Q2, 69.9-77.4 points for Q3, 77.5-86.2 points for Q4, and ≥86.3 points for Q5. Mean±SD was 75.8±13.9 points. Models were conducted using the survey design and sampling weights accounted for.

†P-values for trend across the quintile categories were computed by fitting the ordinal variable of the PHD quintile groups as the exposure variable.
Covariates in each model corresponded to the estimation in each row (crude for estimated averages and crude or multivariable adjusted for odds ratios).

‡Those meeting or exceeding the EAR/DRV/RNI are presented as proportions for the total population and by PHD quintile.

§ Crude and multivariable-adjusted odds ratios (ORs) and corresponding 95% confidence intervals (CIs) were estimated with three models. The first adjusted model included selected covariates: age, sex, ethnicity, occupation, income, region, survey year, season, body-mass index, smoking, alcohol consumption, supplement use, physical activity energy expenditure and chronic health conditions. The second “energy-adjusted model” included total energy intake as a covariate (not fitted when the energy intake was used as the outcome).

¶The odds ratios in the “Total population” column represents the odds ratio per 20-point higher PHD score on a continuous scale from logistic regression models.

NB: for total fat, saturated fat, free sugars and sodium DRV, the percentage refers to those who are consuming less than the DRV for that food group e.g. the higher the percentage, the greater number not exceeding the nutritional guideline.

CI: confidence interval. DRV: dietary reference value. EAR: estimated average requirement. EAR: estimated average requirement. g/Kg: grams per kilogram. Kcal: kilocalories. Mg: milligram. OR: odds ratio. P trend: P-value for trend. PHD: planetary health diet. Q: quintile. Ref: reference category. RE: retinol equivalent. RNI: reference nutrient intake. SD: standard deviation. µg: microgram.

# Supplementary Table 7. Nutritional biomarker status for participants aged ≥15 years in the UK National Diet and Nutrition Survey 2008-2019 (n=4622).

| **Nutritional biomarker concentration** | **Total population (n=4622)** | **PHD Quintile 1***  **(n=794)** | **PHD Quintile 2**  **(n=845)** | **PHD Quintile 3**  **(n=952)** | **PHD Quintile 4**  **(n=960)** | **PHD Quintile 5**  **(n=1071)** | **P trend†** |
| --- | --- | --- | --- | --- | --- | --- | --- |
| **Vitamin B12 (pmol/)*** | 257 (253, 260) | 248 (240, 257) | 249 (242, 256) | 257 (250, 264) | 260 (253, 267) | 265 (258, 273) | <0.001 |
| Biochemical threshold%‡ | 95.3 | 96.2 | 95.7 | 94.7 | 95.9 | 94.4 |  |
| Crude OR (95% CI) | 0.85 (0.65, 1.11)¶ | 1.00 (Ref) | 0.88 (0.50, 1.55) | 0.71 (0.41, 1.23) | 0.92 (0.50, 1.67) | 0.67 (0.39, 1.16) | 0.223 |
| Adjusted OR§ | 0.76 (0.53, 1.07)¶ | 1.00 (Ref) | 0.92 (0.51, 1.66) | 0.77 (0.43, 1.40) | 0.89 (0.48, 1.65) | 0.55 (0.29, 1.04) | 0.092 |
| + energy-adjusted | 0.77 (0.54, 1.09)¶ | 1.00 (Ref) | 0.95 (0.53, 1.72) | 0.81 (0.44, 1.47) | 0.93 (0.51, 1.71) | 0.58 (0.30, 1.10) | 0.119 |
|  |  |  |  |  |  |  |  |
| **HoloTC (pmol/L)*** | 62.6 (61.2, 64.0) | 56.9 (53.8, 60.2) | 62.1 (59.1, 65.3) | 61.4 (58.4, 64.7) | 65.7 (62.5, 69.0) | 64.9 (62.2, 67.7) | <0.001 |
| Biochemical threshold%‡ | 93.7 | 91.0 | 95.0 | 92.9 | 95.3 | 93.6 |  |
| Crude OR (95% CI) | 1.11 (0.82, 1.51)¶ | 1.00 (Ref) | 1.90 (0.89, 4.05) | 1.30 (0.67, 2.53) | 2.01 (0.86, 4.66) | 1.47 (0.78, 2.77) | 0.365 |
| Adjusted OR§ | 1.17 (0.81, 1.68)¶ | 1.00 (Ref) | 1.88 (0.85, 4.14) | 1.55 (0.71, 3.37) | 2.22 (0.97, 5.09) | 1.61 (0.77, 3.35) | 0.295 |
| + energy-adjusted | 1.20 (0.83, 1.74)¶ | 1.00 (Ref) | 1.94 (0.88, 4.26) | 1.65 (0.75, 3.60) | 2.33 (1.04, 5.26) | 1.68 (0.80, 3.54) | 0.255 |
|  |  |  |  |  |  |  |  |
| **25-OHD (nmol/L)*** | 41.3 (40.3, 42.4) | 37.2 (35.0, 39.5) | 40.4 (38.5, 42.5) | 39.7 (37.5, 42.0) | 41.9 (39.9, 43.9) | 45.9 (44.3, 47.6) | <0.001 |
| Biochemical threshold%‡ | 83.4 | 76.3 | 83.6 | 81.2 | 84.3 | 88.9 |  |
| Crude OR (95% CI) | 1.49 (1.28, 1.73)¶ | 1.00 (Ref) | 1.58 (1.13, 2.20) | 1.34 (0.97, 1.86) | 1.67 (1.17, 2.37) | 2.49 (1.77, 3.50) | <0.001 |
| Adjusted OR§ | 1.41 (1.16, 1.71)¶ | 1.00 (Ref) | 1.49 (1.03, 2.15) | 1.25 (0.86, 1.83) | 1.51 (1.01, 2.24) | 2.09 (1.38, 3.16) | 0.001 |
| + energy-adjusted | 1.46 (1.20, 1.78)¶ | 1.00 (Ref) | 1.55 (1.07, 2.25) | 1.32 (0.90, 1.94) | 1.58 (1.06, 2.35) | 2.22 (1.46, 3.36) | 0.001 |
|  |  |  |  |  |  |  |  |
| **Ferritin (µg/L)*** | 67.1 (64.6, 69.7) | 71.5 (65.4, 78.3) | 65.0 (59.8, 70.6) | 69.5 (63.9, 75.7) | 68.0 (62.6, 73.9) | 63.3 (58.8, 68.2) | 0.103 |
| Biochemical threshold%‡ | 92.4 | 94.3 | 92.5 | 91.2 | 92.3 | 92.0 |  |
| Crude OR (95% CI) | 0.92 (0.76, 1.10)¶ | 1.00 (Ref) | 0.74 (0.44, 1.22) | 0.63 (0.39, 1.00) | 0.72 (0.44, 1.17) | 0.69 (0.44, 1.10) | 0.213 |
| Adjusted OR§ | 0.95 (0.77, 1.18)¶ | 1.00 (Ref) | 0.75 (0.45, 1.24) | 0.65 (0.40, 1.05) | 0.78 (0.47, 1.29) | 0.69 (0.42, 1.13) | 0.298 |
| + energy-adjusted | 0.95 (0.77, 1.17)¶ | 1.00 (Ref) | 0.74 (0.45, 1.23) | 0.64 (0.40, 1.02) | 0.77 (0.46, 1.27) | 0.68 (0.42, 1.10) | 0.273 |
|  |  |  |  |  |  |  |  |
| **Zinc (µmol/L)*** | 13.4 (13.3, 13.6) | 13.6 (13.3, 13.9) | 13.6 (13.4, 13.9) | 13.5 (13.2, 13.7) | 13.4 (13.2, 13.6) | 13.3 (13.1 , 13.5) | 0.01 |
| Beta coefficient (95% CI) | - | *-* | *-* | *-* | *-* | *-* |  |
| Crude | -1.61 (-2.61, -0.60) | *-* | *-* | *-* | *-* | *-* | 0.002 |
| Adjusted | -0.63 (-1.68, 0.43) | *-* | *-* | *-* | *-* | *-* | 0.24 |
| +energy-adjusted | -0.54 (-1.59, 0.52) | *-* | *-* | *-* | *-* | *-* | 0.32 |
|  |  |  |  |  |  |  |  |
| **Riboflavin (EGRAC)*** | 1.4 (1.3, 1.4) | 1.4 (1.4, 1.4) | 1.4 (1.4, 1.4) | 1.3 (1.3, 1.4) | 1.3 (1.3, 1.4) | 1.3 (1.3, 1.3) | <0.001 |
| Biochemical threshold%‡ | 39.7 | 28.6 | 34.7 | 44.1 | 42.3 | 44.5 |  |
| Crude OR (95% CI) | 1.33 (1.19, 1.49)¶ | 1.00 (Ref) | 1.33 (0.99, 1.79) | 1.96 (1.49, 2.56) | 1.83 (1.41, 2.38) | 2.01 (1.54, 2.63) | <0.001 |
| Adjusted OR§ | 1.01 (0.88, 1.16)¶ | 1.00 (Ref) | 1.26 (0.93, 1.72) | 1.56 (1.15, 2.13) | 1.37 (1.02, 1.85) | 1.23 (0.91, 1.67) | 0.338 |
| + energy-adjusted | 1.02 (0.89, 1.18)¶ | 1.00 (Ref) | 1.28 (0.93, 1.75) | 1.60 (1.18, 2.17) | 1.39 (1.03, 1.89) | 1.25 (0.92, 1.69) | 0.296 |
|  |  |  |  |  |  |  |  |
| **Folate (nmol/L)*** | 15.9 (15.6, 16.3) | 12.2 (11.4, 13.0) | 13.5 (12.9, 14.2) | 15.4 (14.7, 16.2) | 17.7 (16.9, 18.5) | 20.1 (19.3, 20.9) | <0.001 |
| Biochemical threshold%‡ | 61.4 | 38.2 | 49.6 | 60.9 | 69.5 | 78.3 |  |
| Crude OR (95% CI) | 2.49 (2.19, 2.83)¶ | 1.00 (Ref) | 1.60 (1.20, 2.12) | 2.52 (1.91, 3.32) | 3.69 (2.79, 4.87) | 5.85 (4.47, 7.67) | <0.001 |
| Adjusted OR§ | 2.14 (1.86, 2.47)¶ | 1.00 (Ref) | 1.42 (1.06, 1.90) | 2.11 (1.57, 2.82) | 2.78 (2.05, 3.77) | 4.40 (3.25, 5.96) | <0.001 |
| + energy-adjusted | 2.17 (1.88, 2.50)¶ | 1.00 (Ref) | 1.43 (1.07, 1.93) | 2.14 (1.60, 2.88) | 2.83 (2.08, 3.85) | 4.49 (3.31, 6.10) | <0.001 |
|  |  |  |  |  |  |  |  |
| **Urinary Iodine (g/L)*** | 109 (106, 113) | 102 (95, 110) | 118 (107, 130) | 117 (108, 127) | 111 (105, 118) | 102 (95, 109) | 0.272 |
| Biochemical threshold%‡ | 57.3 | 54.7 | 61.8 | 56.3 | 61.2 | 53.6 |  |
| Crude OR (95% CI) | 0.95 (0.84, 1.07)¶ | 1.00 (Ref) | 1.34 (1.00, 1.79) | 1.07 (0.81, 1.40) | 1.31 (0.98, 1.74) | 0.96 (0.74, 1.24) | 0.462 |
| Adjusted OR§ | 0.90 (0.78, 1.03)¶ | 1.00 (Ref) | 1.34 (0.98, 1.82) | 0.98 (0.73, 1.32) | 1.20 (0.88, 1.65) | 0.85 (0.63, 1.15) | 0.128 |
| + energy-adjusted | 0.90 (0.78, 1.03)¶ | 1.00 (Ref) | 1.35 (1.00, 1.84) | 1.00 (0.74, 1.35) | 1.22 (0.89, 1.68) | 0.86 (0.63, 1.16) | 0.132 |
|  |  |  |  |  |  |  |  |

* Geometric mean and 95% confidence intervals are reported. The score ranges of the five categories were <62.0 points for Q1, 62.0-69.8 points for Q2, 69.9-77.4 points for Q3, 77.5-86.2 points for Q4, and ≥86.3 points for Q5. Mean±SD was 75.8±13.9 points. Models were conducted using the survey design and sampling weights accounted for.

†P-values for trend across the quintile categories were computed by fitting the ordinal variable of the PHD quintile groups as the exposure variable. Covariates in each model corresponded to the estimation in each row (crude for estimated averages and crude or multivariable adjusted for odds ratios).

‡Those meeting or exceeding the biochemical threshold are presented as proportions for the total population and by PHD quintile.

§ Crude and multivariable-adjusted odds ratios (ORs) and corresponding 95% confidence intervals (CIs) were estimated with three models. The first adjusted model included selected covariates: age, sex, ethnicity, occupation, income, region, survey year, season, body-mass index, smoking, alcohol consumption, supplement use, physical activity energy expenditure and chronic health conditions. The second “energy-adjusted model” included total energy intake as a covariate.

¶The odds ratios in the “Total population” column represents the odds ratio per 20-point higher PHD score on a continuous scale from logistic regression models.

For circulating zinc concentrations, there is no NDNS-established biomarker threshold for sufficiency, as such, the beta-coefficients and p-values per 20-point higher PHD score on a continuous scale are reported rather than odds ratios. The beta-coefficient (95%CI) was back-transformed in the following way: 100 x (exp(ln β-coefficient)-1).

25-OHD: 25-hydroxyvitamin D. β: beta. CI: confidence interval. DRV: Dietary reference value. EAR: Estimated average requirement. EGRAC: Erythrocyte Glutathione Reductase Activation Coefficient. g/L: grams per liter. HoloTC: holotranscobalamin. Kcal: kilocalories. Kg: kilogram. Mg: milligram. NDNS: National Diet and Nutrition Survey. nmol/L: nanomoles per liter. OR: odds ratio. P trend: P-value for trend. PHD: planetary health diet. pmol/L: picomoles per liter. Q: quintile. RE: retinol equivalent. Ref: reference category. RNI: Reference nutrient intake. SD: standard deviation. µg: microgram. µg/L: micrograms per liter.

# Supplementary Table 8. Intake of selected nutrients per day for adolescents aged 15-18 year olds in the UK National Diet and Nutrition Survey 2008-2019 (n=1672).

| **Nutrient intake per day** | **Total population (n=1672)** | **PHD Quintile 1***  **(n=335)** | **PHD Quintile 2**  **(n=334)** | **PHD Quintile 3**  **(n=335)** | **PHD Quintile 4**  **(n=334)** | **PHD Quintile 5**  **(n=334)** | **P trend†** |
| --- | --- | --- | --- | --- | --- | --- | --- |
| **Vitamin B12, µg*** | 3.7 (3.6, 3.9) | 4.2 (3.9, 4.6) | 3.6 (3.3, 3.9) | 3.8 (3.5, 4.1) | 3.5 (3.2, 3.7) | 3.6 (3.3, 4.0) | 0.023 |
| RNI%‡ | 94.5 | 97.4 | 94.2 | 94.1 | 94.2 | 93.0 |  |
| Crude OR (95% CI) | 0.67 (0.44, 1.00)¶ | 1.00 (Ref) | 0.44 (0.19, 1.05) | 0.43 (0.19, 1.01) | 0.44 (0.18, 1.06) | 0.36 (0.16, 0.82) | 0.071 |
| Adjusted OR§ | 0.64 (0.39, 1.04)¶ | 1.00 (Ref) | 0.64 (0.27, 1.51) | 0.57 (0.23, 1.44) | 0.64 (0.24, 1.68) | 0.41 (0.15, 1.07) | 0.122 |
| + energy-adjusted | 0.82 (0.43, 1.55)¶ | 1.00 (Ref) | 1.11 (0.47, 2.65) | 1.22 (0.46, 3.19) | 1.52 (0.61, 3.77) | 0.92 (0.33, 2.58) | 0.941 |
|  |  |  |  |  |  |  |  |
| **Vitamin D, µg*** | 1.74 (1.66, 1.83) | 2.01 (1.86, 2.17) | 1.62 (1.45, 1.80) | 1.72 (1.53, 1.93) | 1.63 (1.48, 1.81) | 1.78 (1.58, 2.02) | 0.272 |
| RNI%‡ | 0.3 | 0 | 0 | 0.6 | 0 | 0.8 |  |
| Crude OR (95% CI) | - | - | - | - | - | - | - |
| Adjusted OR§ | - | - | - | - | - | - | - |
| + energy-adjusted | - | - | - | - | - | - | - |
|  |  |  |  |  |  |  |  |
| **Calcium, mg*** | 703 (684, 723) | 745 (699, 793) | 678 (641, 718) | 708 (662, 757) | 663 (626, 702) | 727 (683, 774) | 0.608 |
| RNI%‡ | 27.3 | 27.1 | 21.6 | 29.8 | 20.8 | 36.2 |  |
| Crude OR (95% CI) | 1.26 (0.99, 1.60)¶ | 1.00 (Ref) | 0.74 (0.47, 1.17) | 1.14 (0.75, 1.75) | 0.71 (0.46, 1.10) | 1.53 (1.01, 2.32) | 0.056 |
| Adjusted OR§ | 1.38 (1.06, 1.80)¶ | 1.00 (Ref) | 0.73 (0.45, 1.20) | 1.27 (0.81, 1.99) | 0.79 (0.49, 1.28) | 1.76 (1.09, 2.84) | 0.025 |
| + energy-adjusted | 2.22 (1.64, 3.01)¶ | 1.00 (Ref) | 1.18 (0.67, 2.09) | 2.45 (1.39, 4.31) | 1.49 (0.84, 2.65) | 5.21 (2.94, 9.23) | <0.001 |
|  |  |  |  |  |  |  |  |
| **Iron, mg*** | 9 (9, 9) | 9 (9, 10) | 8 (8, 9) | 9 (8, 9) | 9 (8, 9) | 9 (9, 10) | 0.168 |
| RNI%‡ | 21.3 | 28.1 | 19.1 | 18.8 | 18.7 | 22.6 |  |
| Crude OR (95% CI) | 0.94 (0.69, 1.28)¶ | 1.00 (Ref) | 0.60 (0.36, 1.01) | 0.59 (0.36, 0.98) | 0.59 (0.36, 0.96) | 0.75 (0.45, 1.24) | 0.358 |
| Adjusted OR§ | 1.84 (1.32, 2.56)¶ | 1.00 (Ref) | 1.02 (0.56, 1.86) | 1.09 (0.60, 1.96) | 1.23 (0.68, 2.22) | 2.28 (1.25, 4.17) | 0.013 |
| + energy-adjusted | 2.78 (1.83, 4.22)¶ | 1.00 (Ref) | 1.72 (0.84, 3.54) | 1.37 (0.69, 2.72) | 1.97 (0.94, 4.12) | 4.76 (2.33, 9.73) | <0.001 |
|  |  |  |  |  |  |  |  |
| **Zinc, mg*** | 7.06 (6.88, 7.24) | 8.03 (7.61, 8.48) | 6.77 (6.41, 7.15) | 6.99 (6.56, 7.45) | 6.69 (6.33, 7.08) | 6.98 (6.60, 7.38) | 0.003 |
| RNI%‡ | 36.6 | 41.8 | 34.5 | 36.3 | 31.5 | 39.1 |  |
| Crude OR (95% CI) | 0.97 (0.77, 1.23)¶ | 1.00 (Ref) | 0.73 (0.48, 1.12) | 0.79 (0.52, 1.21) | 0.64 (0.42, 0.97) | 0.89 (0.59, 1.36) | 0.551 |
| Adjusted OR§ | 0.99 (0.76, 1.27)¶ | 1.00 (Ref) | 0.73 (0.46, 1.15) | 0.85 (0.54, 1.35) | 0.68 (0.44, 1.07) | 0.92 (0.58, 1.46) | 0.718 |
| + energy-adjusted | 1.44 (1.07, 1.94)¶ | 1.00 (Ref) | 1.19 (0.69, 2.03) | 1.51 (0.89, 2.56) | 1.26 (0.72, 2.20) | 2.16 (1.24, 3.74) | 0.012 |

* Geometric mean and 95% confidence intervals are reported. The score ranges of the five categories were <62.0 points for Q1, 62.0-69.8 points for Q2, 69.9-77.4 points for Q3, 77.5-86.2 points for Q4, and ≥86.3 points for Q5. Mean±SD was 75.8±13.9 points. Models were conducted using the survey design and sampling weights accounted for.

†P-values for trend across the quintile categories were computed by fitting the ordinal variable of the PHD quintile groups as the exposure variable. Covariates in each model corresponded to the estimation in each row (crude for estimated averages and crude or multivariable adjusted for odds ratios).

‡Those meeting or exceeding the EAR/DRV/RNI are presented as proportions for the total population and by PHD quintile.

§ Crude and multivariable-adjusted odds ratios (ORs) and corresponding 95% confidence intervals (CIs) were estimated with three models. The first adjusted model included selected covariates: age, sex, ethnicity, occupation, income, region, survey year, season, body-mass index, smoking, alcohol consumption, supplement use, physical activity energy expenditure and chronic health conditions. The second “energy-adjusted model” included total energy intake as a covariate (not fitted when the energy intake was used as the outcome). For vitamin D intake, there were insufficient participants reaching the RNI and so logistic regression models were not fitted.

¶The odds ratios in the “Total population” column represents the odds ratio per 20-point higher PHD score on a continuous scale from logistic regression models.
CI: confidence interval. mg: milligram. OR: odds ratio. P trend: P-value for trend. PHD: planetary health diet. Q: quintile. Ref: reference category. RNI: reference nutrient intake. SD: standard deviation. µg: microgram.

# Supplementary Table 9. Intake of selected nutrients per day for adults aged 19-64 years of age in the UK National Diet and Nutrition Survey 2008-2019 (n=6136).

| **Nutrient intake per day** | **Total population  (n=6136)** | **PHD Quintile 1***  **(n=1228)** | **PHD Quintile 2**  **(n=1227)** | **PHD Quintile 3**  **(n=1227)** | **PHD Quintile 4**  **(n=1227)** | **PHD Quintile 5**  **(n=1227)** | **P trend†** |
| --- | --- | --- | --- | --- | --- | --- | --- |
| **Vitamin B12, µg*** | 4.34 (4.26, 4.42) | 4.40 (4.22, 4.59) | 4.29 (4.13, 4.45) | 4.20 (4.03, 4.38) | 4.46 (4.27, 4.67) | 4.35 (4.16, 4.55) | 0.763 |
| RNI%‡ | 95.8 | 96.9 | 96.6 | 94.3 | 95.3 | 95.9 |  |
| Crude OR (95% CI) | 0.85 (0.68, 1.06)¶ | 1.00 (Ref) | 0.91 (0.52, 1.58) | 0.54 (0.32, 0.89) | 0.65 (0.36, 1.15) | 0.74 (0.43, 1.30) | 0.147 |
| Adjusted OR§ | 0.77 (0.59, 0.99)¶ | 1.00 (Ref) | 0.72 (0.40, 1.30) | 0.44 (0.25, 0.78) | 0.48 (0.25, 0.90) | 0.55 (0.29, 1.04) | 0.029 |
| + energy-adjusted | 0.89 (0.64, 1.24)¶ | 1.00 (Ref) | 0.87 (0.45, 1.70) | 0.59 (0.31, 1.11) | 0.65 (0.33, 1.28) | 0.78 (0.37, 1.63) | 0.420 |
|  |  |  |  |  |  |  |  |
| **Vitamin D, µg*** | 2.19 (2.13, 2.24) | 2.10 (2.00, 2.20) | 2.09 (1.99, 2.19) | 2.04 (1.93, 2.16) | 2.27 (2.14, 2.40) | 2.43 (2.29, 2.58) | <0.001 |
| RNI%‡ | 1.3 | 0.07 | 1.2 | 0.9 | 1.4 | 2.9 |  |
| Crude OR (95% CI) | 2.8 (1.9, 4.4)¶ | 1.00 (Ref) | 15.8 (4.4, 57.2) | 12.3 (3.5, 43.9) | 18.1 (5.4, 60.1) | 37.7 (12.0, 118.2) | <0.001 |
| Adjusted OR§ | 2.9 (1.7, 4.9)¶ | 1.00 (Ref) | 18.9 (4.5, 79.6) | 14.9 (3.6, 62.0) | 23.9 (6.6, 87.5) | 36.5 (9.4, 142.3) | 0.001 |
| + energy-adjusted | 3.0 (1.7, 5.3)¶ | 1.00 (Ref) | 36.0 (5.4, 239.9) | 24.2 (3.9, 148.7) | 47.2 (7.6, 291.3) | 67.3 (10.0, 449.9) | 0.001 |
|  |  |  |  |  |  |  |  |
| **Calcium, mg*** | 751 (741, 761) | 725 (702, 749) | 745 (724, 767) | 738 (717, 760) | 757 (735, 780) | 787 (766, 809) | <0.001 |
| RNI%‡ | 59.8 | 56.1 | 58.3 | 58.1 | 61.2 | 64.7 |  |
| Crude OR (95% CI) | 1.19 (1.08, 1.31)¶ | 1.00 (Ref) | 1.10 (0.89, 1.34) | 1.08 (0.88, 1.33) | 1.24 (1.00, 1.53) | 1.43 (1.16, 1.76) | <0.001 |
| Adjusted OR§ | 1.25 (1.11, 1.40)¶ | 1.00 (Ref) | 1.12 (0.89, 1.41) | 1.14 (0.90, 1.44) | 1.32 (1.03, 1.69) | 1.53 (1.19, 1.98) | 0.001 |
| + energy-adjusted | 1.66 (1.43, 1.92)¶ | 1.00 (Ref) | 1.59 (1.20, 2.10) | 1.80 (1.35, 2.41) | 2.16 (1.62, 2.87) | 2.71 (2.00, 3.68) | <0.001 |
|  |  |  |  |  |  |  |  |
| **Iron, mg*** | 10 (10, 10) | 9 (9, 9) | 9 (9, 9) | 10 (9, 10) | 10 (10, 11) | 11 (11, 11) | <0.001 |
| RNI%‡ | 49.3 | 44.7 | 42.8 | 49.4 | 51.3 | 57.3 |  |
| Crude OR (95% CI) | 1.32 (1.20, 1.45)¶ | 1.00 (Ref) | 0.92 (0.75, 1.14) | 1.21 (0.98, 1.48) | 1.30 (1.06, 1.60) | 1.66 (1.35, 2.04) | <0.001 |
| Adjusted OR§ | 1.71 (1.49, 1.98)¶ | 1.00 (Ref) | 1.05 (0.79, 1.41) | 1.51 (1.12, 2.03) | 1.92 (1.42, 2.61) | 2.72 (2.00, 3.70) | <0.001 |
| + energy-adjusted | 2.27 (1.90, 2.73)¶ | 1.00 (Ref) | 1.43 (1.02, 1.99) | 2.23 (1.61, 3.08) | 3.05 (2.11, 4.42) | 4.71 (3.22, 6.90) | <0.001 |
|  |  |  |  |  |  |  |  |
| **Zinc, mg*** | 8.06 (7.96, 8.16) | 8.19 (7.95, 8.43) | 7.91 (7.70, 8.12) | 7.91 (7.69, 8.12) | 8.07 (7.86, 8.28) | 8.21 (8.02, 8.41) | 0.513 |
| RNI%‡ | 52.0 | 48.8 | 51.6 | 48.9 | 52.5 | 57.8 |  |
| Crude OR (95% CI) | 1.20 (1.09, 1.32)¶ | 1.00 (Ref) | 1.12 (0.91, 1.38) | 1.00 (0.82, 1.23) | 1.16 (0.95, 1.42) | 1.43 (1.17, 1.76) | 0.001 |
| Adjusted OR§ | 1.03 (0.92, 1.16)¶ | 1.00 (Ref) | 0.98 (0.78, 1.23) | 0.84 (0.66, 1.05) | 0.84 (0.67, 1.06) | 1.06 (0.82, 1.36) | 0.969 |
| + energy-adjusted | 1.20 (1.04, 1.37)¶ | 1.00 (Ref) | 1.23 (0.93, 1.64) | 1.06 (0.79, 1.42) | 1.07 (0.81, 1.43) | 1.45 (1.07, 1.96) | 0.067 |

* Geometric mean and 95% confidence intervals are reported. The score ranges of the five categories were <62.0 points for Q1, 62.0-69.8 points for Q2, 69.9-77.4 points for Q3, 77.5-86.2 points for Q4, and ≥86.3 points for Q5. Mean±SD was 75.8±13.9 points. Models were conducted using the survey design and sampling weights accounted for.

†P-values for trend across the quintile categories were computed by fitting the ordinal variable of the PHD quintile groups as the exposure variable. Covariates in each model corresponded to the estimation in each row (crude for estimated averages and crude or multivariable adjusted for odds ratios).

‡Those meeting or exceeding the EAR/DRV/RNI are presented as proportions for the total population and by PHD quintile.

§ Crude and multivariable-adjusted odds ratios (ORs) and corresponding 95% confidence intervals (CIs) were estimated with three models. The first adjusted model included selected covariates: age, sex, ethnicity, occupation, income, region, survey year, season, body-mass index, smoking, alcohol consumption, supplement use, physical activity energy expenditure and chronic health conditions. The second “energy-adjusted model” included total energy intake as a covariate (not fitted when the energy intake was used as the outcome).

¶The odds ratios in the “Total population” column represents the odds ratio per 20-point higher PHD score on a continuous scale from logistic regression models.

CI: confidence interval. mg: milligram. OR: odds ratio. P trend: P-value for trend. PHD: planetary health diet. Q: quintile. Ref: reference category. RNI: reference nutrient intake. SD: standard deviation. µg: microgram.

# Supplementary Table 10. Intake of selected nutrients per day for adults aged 65 years of age and over in the UK National Diet and Nutrition Survey 2008-2019 (n=1863).

| **Nutrient intake per day** | **Total population (n=1863)** | **PHD Quintile 1***  **(n=373)** | **PHD Quintile 2**  **(n=373)** | **PHD Quintile 3**  **(n=372)** | **PHD Quintile 4**  **(n=373)** | **PHD Quintile 5**  **(n=372)** | **P trend†** |
| --- | --- | --- | --- | --- | --- | --- | --- |
| **Vitamin B12, µg*** | 5.0 (4.9, 5.2) | 4.7 (4.4, 5.1) | 4.9 (4.6, 5.3) | 4.9 (4.6, 5.3) | 5.2 (4.8, 5.7) | 5.3 (4.9, 5.8) | 0.009 |
| RNI%‡ | 98.0 | 98.8 | 98.5 | 99.4 | 96.2 | 97.2 |  |
| Crude OR (95% CI) | 0.38 (0.15, 0.99)¶ | 1.00 (Ref) | 0.81 (0.17, 3.88) | 2.07 (0.47, 9.23) | 0.32 (0.09, 1.10) | 0.44 (0.11, 1.72) | 0.089 |
| Adjusted OR§ | 0.38 (0.12, 1.23)¶ | 1.00 (Ref) | 0.66 (0.14, 3.14) | 0.74 (0.12, 4.43) | 0.30 (0.06, 1.50) | 0.24 (0.04, 1.47) | 0.141 |
| + energy-adjusted | 0.18 (0.06, 0.58)¶ | 1.00 (Ref) | 1.04 (0.18, 5.88) | 0.67 (0.12, 3.68) | 0.40 (0.07, 2.28) | 0.14 (0.02, 0.88) | 0.027 |
|  |  |  |  |  |  |  |  |
| **Vitamin D, µg*** | 2.62 (2.51, 2.73) | 2.26 (2.10, 2.44) | 2.43 (2.24, 2.64) | 2.48 (2.28, 2.70) | 2.64 (2.34, 2.98) | 3.27 (2.96, 3.60) | <0.001 |
| RNI%‡ | 1.3 | 0 | 0.6 | 0.5 | 3.3 | 1.8 |  |
| Crude OR (95% CI) | 2.7 (1.7, 4.2)¶ | 1.00 (Ref) | 0.3 (0.09, 1.3) | 0.3 (0.06, 1.5) | 1.9 (0.7, 5.3) | - | <0.001 |
| Adjusted OR§ | 5.4 (2.5, 11.7)¶ | 1.00 (Ref) | 0.2 (0.03, 1.04) | 0.06 (0.006, 0.67) | 1.70 (0.52, 5.5) | - | <0.001 |
| + energy-adjusted | 6.3 (2.7, 14.7)¶ | 1.00 (Ref) | 0.2 (0.03, 0.83) | 0.06 (0.007, 0.53) | 1.59 (0.48, 5.2) | - | <0.001 |
|  |  |  |  |  |  |  |  |
| **Calcium, mg*** | 767 (751, 784) | 720 (683, 760) | 767 (731, 805) | 763 (729, 800) | 766 (728, 806) | 813 (785, 843) | 0.001 |
| RNI%‡ | 62.7 | 57.3 | 60.2 | 61.6 | 61.8 | 70.9 |  |
| Crude OR (95% CI) | 1.39 (1.15, 1.66)¶ | 1.00 (Ref) | 1.13 (0.78, 1.63) | 1.19 (0.82, 1.73) | 1.21 (0.83, 1.76) | 1.82 (1.26, 2.63) | 0.002 |
| Adjusted OR§ | 1.46 (1.15, 1.85)¶ | 1.00 (Ref) | 1.17 (0.76, 1.79) | 1.19 (0.76, 1.86) | 1.28 (0.82, 1.99) | 1.84 (1.17, 2.90) | 0.009 |
| + energy-adjusted | 1.85 (1.39, 2.47)¶ | 1.00 (Ref) | 1.47 (0.90, 2.41) | 1.37 (0.82, 2.29) | 1.75 (1.03, 2.97) | 2.74 (1.61, 4.68) | <0.001 |
|  |  |  |  |  |  |  |  |
| **Iron, mg*** | 9 (9, 10) | 8 (8, 9) | 9 (8, 9) | 9 (9, 10) | 10 (10, 10) | 11 (10, 11) | <0.001 |
| RNI%‡ | 59.8 | 49.0 | 47.7 | 59.2 | 66.8 | 73.3 |  |
| Crude OR (95% CI) | 1.94 (1.60, 2.36)¶ | 1.00 (Ref) | 0.95 (0.66, 1.37) | 1.51 (1.05, 2.18) | 2.10 (1.44, 3.06) | 2.86 (1.96, 4.18) | <0.001 |
| Adjusted OR§ | 1.93 (1.51, 2.45)¶ | 1.00 (Ref) | 1.21 (0.78, 1.89) | 1.82 (1.18, 2.82) | 2.30 (1.48, 3.58) | 2.95 (1.84, 4.71) | <0.001 |
| + energy-adjusted | 2.59 (1.96, 3.43)¶ | 1.00 (Ref) | 1.49 (0.89, 2.51) | 2.49 (1.50, 4.13) | 3.61 (2.15, 6.05) | 4.81 (2.85, 8.10) | <0.001 |
|  |  |  |  |  |  |  |  |
| **Zinc, mg*** | 7.7 (7.5, 7.8) | 7.7 (7.4, 8.1) | 7.4 (7.2, 7.7) | 7.5 (7.2, 7.8) | 7.7 (7.4, 8.0) | 8.0 (7.7, 8.3) | 0.103 |
| RNI%‡ | 46.2 | 44.2 | 38.2 | 46.2 | 48.6 | 52.6 |  |
| Crude OR (95% CI) | 1.34 (1.12, 1.62)¶ | 1.00 (Ref) | 0.78 (0.53, 1.15) | 1.09 (0.74, 1.59) | 1.19 (0.81, 1.76) | 1.40 (0.96, 2.04) | 0.009 |
| Adjusted OR§ | 1.02 (0.81, 1.29)¶ | 1.00 (Ref) | 0.78 (0.49, 1.22) | 0.84 (0.54, 1.31) | 0.90 (0.57, 1.43) | 0.89 (0.56, 1.41) | 0.927 |
| + energy-adjusted | 1.15 (0.87, 1.53)¶ | 1.00 (Ref) | 0.88 (0.52, 1.48) | 0.87 (0.50, 1.52) | 1.08 (0.64, 1.84) | 1.04 (0.61, 1.78) | 0.605 |

* Geometric mean and 95% confidence intervals are reported. The score ranges of the five categories were <62.0 points for Q1, 62.0-69.8 points for Q2, 69.9-77.4 points for Q3, 77.5-86.2 points for Q4, and ≥86.3 points for Q5. Mean±SD was 75.8±13.9 points. Models were conducted using the survey design and sampling weights accounted for.

†P-values for trend across the quintile categories were computed by fitting the ordinal variable of the PHD quintile groups as the exposure variable. Covariates in each model corresponded to the estimation in each row (crude for estimated averages and crude or multivariable adjusted for odds ratios).

‡Those meeting or exceeding the EAR/DRV/RNI are presented as proportions for the total population and by PHD quintile.

§ Crude and multivariable-adjusted odds ratios (ORs) and corresponding 95% confidence intervals (CIs) were estimated with three models. The first adjusted model included selected covariates: age, sex, ethnicity, occupation, income, region, survey year, season, body-mass index, smoking, alcohol consumption, supplement use, physical activity energy expenditure and chronic health conditions. The second “energy-adjusted model” included total energy intake as a covariate (not fitted when the energy intake was used as the outcome). For vitamin D intake, there were insufficient participants reaching the RNI in Q5 and so logistic regression models were not fitted for Q5. ¶The odds ratios in the “Total population” column represents the odds ratio per 20-point higher PHD score on a continuous scale from logistic regression models. CI: confidence interval. mg: milligram. OR: odds ratio. P trend: P-value for trend. PHD: planetary health diet. Q: quintile. Ref: reference category. RNI: reference nutrient intake. SD: standard deviation. µg: microgram.

# Supplementary Table 11. Intake of selected nutrients per day for women of reproductive age (15-49 years) in the UK National Diet and Nutrition Survey 2008-2019 (n=3350).

| **Nutrient intake per day** | **Total population (n=3350)** | **PHD Quintile 1***  **(n=670)** | **PHD Quintile 2**  **(n=670)** | **PHD Quintile 3**  **(n=670)** | **PHD Quintile 4**  **(n=670)** | **PHD Quintile 5**  **(n=670)** | **P trend†** |
| --- | --- | --- | --- | --- | --- | --- | --- |
| **Vitamin B12, µg*** | 3.6 (3.5, 3.7) | 3.6 (3.4, 3.8) | 3.5 (3.3, 3.7) | 3.7 (3.5, 3.9) | 3.6 (3.4, 3.8) | 3.7 (3.5, 3.9) | 0.431 |
| RNI%‡ | 93.6 | 95.4 | 94.5 | 94.0 | 91.8 | 93.0 |  |
| Crude OR (95% CI) | 0.76 (0.58, 1.00)¶ | 1.00 (Ref) | 0.82 (0.44, 1.52) | 0.74 (0.40, 1.37) | 0.53 (0.29, 0.98) | 0.63 (0.33, 1.20) | 0.063 |
| Adjusted OR§ | 0.72 (0.53, 0.98)¶ | 1.00 (Ref) | 0.72 (0.37, 1.41) | 0.65 (0.32, 1.31) | 0.46 (0.23, 0.93) | 0.57 (0.28, 1.16) | 0.065 |
| + energy-adjusted | 0.82 (0.54, 1.23)¶ | 1.00 (Ref) | 0.92 (0.46, 1.83) | 1.00 (0.46, 2.14) | 0.68 (0.33, 1.41) | 0.84 (0.38, 1.87) | 0.495 |
|  |  |  |  |  |  |  |  |
| **Vitamin D, µg*** | 1.71 (1.60, 1.83) | 1.71 (1.60, 1.83) | 1.63 (1.51, 1.76) | 1.81 (1.68, 1.96) | 1.83 (1.68, 1.99) | 2.11 (1.94, 2.28) | <0.001 |
| RNI%‡ | 0.8 | , | 0.3 (0.06, 1.6) | 1.4 (0.4, 4.1) | 0.2 (0.02, 1.2) | 1.9 (0.7, 4.9) |  |
| Crude OR (95% CI) | 2.8 (1.3, 6.2)¶ | 1.00 (Ref) | 0.17 (0.03, 1.12) | 0.69 (0.16, 3.1) | 0.09 (0.01, 0.79) | - | 0.049 |
| Adjusted OR§ | 1.5 (0.5, 4.3)¶ | 1.00 (Ref) | 0.63 (0.07, 5.9) | 1.9 (0.36, 9.7) | 0.21 (0.01, 3.4) | - | 0.55 |
| + energy-adjusted | 1.6 (0.6, 4.4)¶ | 1.00 (Ref) | 0.46 (0.05, 4.2) | 1.5 (0.38, 6.2) | 0.19 (0.01, 3.3) | - | 0.29 |
|  |  |  |  |  |  |  |  |
| **Calcium, mg*** | 662 (651, 674) | 635 (608, 662) | 646 (622, 671) | 658 (630, 686) | 668 (640, 698) | 693 (669, 718) | 0.001 |
| RNI%‡ | 46.2 | 43.7 | 38.8 | 44.9 | 47.9 | 52.8 |  |
| Crude OR (95% CI) | 1.32 (1.14, 1.54)¶ | 1.00 (Ref) | 0.82 (0.59, 1.13) | 1.05 (0.76, 1.44) | 1.18 (0.87, 1.62) | 1.44 (1.06, 1.97) | 0.001 |
| Adjusted OR§ | 1.18 (0.98, 1.42)¶ | 1.00 (Ref) | 0.79 (0.55, 1.12) | 0.93 (0.65, 1.32) | 1.03 (0.72, 1.47) | 1.18 (0.81, 1.70) | 0.124 |
| + energy-adjusted | 1.69 (1.35, 2.12)¶ | 1.00 (Ref) | 1.24 (0.82, 1.88) | 1.67 (1.08, 2.58) | 1.92 (1.25, 2.95) | 2.67 (1.71, 4.16) | <0.001 |
|  |  |  |  |  |  |  |  |
| **Iron, mg*** | 9 (9, 9) | 8 (8, 8) | 8 (8, 8) | 9 (8, 9) | 9 (9, 9) | 10 (10, 10) | <0.001 |
| RNI%‡ | 4.8 | 1.6 | 3.2 | 4.9 | 5.5 | 7.6 |  |
| Crude OR (95% CI) | 2.02 (1.45, 2.81)¶ | 1.00 (Ref) | 2.03 (0.82, 5.06) | 3.16 (1.31, 7.63) | 3.61 (1.63, 8.02) | 5.08 (2.37, 10.88) | <0.001 |
| Adjusted OR§ | 1.66 (1.13, 2.44)¶ | 1.00 (Ref) | 1.59 (0.61, 4.15) | 2.61 (1.01, 6.77) | 2.98 (1.24, 7.18) | 3.22 (1.37, 7.53) | 0.004 |
| + energy-adjusted | 2.14 (1.41, 3.26)¶ | 1.00 (Ref) | 2.58 (0.85, 7.76) | 4.27 (1.47, 12.41) | 5.65 (1.95, 16.43) | 7.04 (2.50, 19.81) | <0.001 |
|  |  |  |  |  |  |  |  |
| **Zinc, mg*** | 6.94 (6.83, 7.06) | 6.77 (6.50, 7.05) | 6.72 (6.48, 6.96) | 6.90 (6.64, 7.16) | 6.97 (6.71, 7.23) | 7.26 (7.04, 7.48) | 0.002 |
| RNI%‡ | 52.6 | 50.6 | 52.3 | 49.9 | 52.7 | 56.4 |  |
| Crude OR (95% CI) | 1.15 (0.99, 1.33)¶ | 1.00 (Ref) | 1.07 (0.78, 1.46) | 0.97 (0.71, 1.33) | 1.09 (0.80, 1.48) | 1.26 (0.93, 1.71) | 0.140 |
| Adjusted OR§ | 0.96 (0.80, 1.15)¶ | 1.00 (Ref) | 1.05 (0.75, 1.48) | 0.88 (0.63, 1.25) | 0.87 (0.60, 1.25) | 0.91 (0.62, 1.33) | 0.416 |
| + energy-adjusted | 1.26 (1.02, 1.55)¶ | 1.00 (Ref) | 1.71 (1.13, 2.59) | 1.51 (0.99, 2.31) | 1.49 (0.97, 2.28) | 1.81 (1.16, 2.80) | 0.053 |

* Geometric mean and 95% confidence intervals are reported. The score ranges of the five categories were <62.0 points for Q1, 62.0-69.8 points for Q2, 69.9-77.4 points for Q3, 77.5-86.2 points for Q4, and ≥86.3 points for Q5. Mean±SD was 75.8±13.9 points. Models were conducted using the survey design and sampling weights accounted for.

†P-values for trend across the quintile categories were computed by fitting the ordinal variable of the PHD quintile groups as the exposure variable. Covariates in each model corresponded to the estimation in each row (crude for estimated averages and crude or multivariable adjusted for odds ratios).

‡Those meeting or exceeding the EAR/DRV/RNI are presented as proportions for the total population and by PHD quintile.

§ Crude and multivariable-adjusted odds ratios (ORs) and corresponding 95% confidence intervals (CIs) were estimated with three models. The first adjusted model included selected covariates: age, sex, ethnicity, occupation, income, region, survey year, season, body-mass index, smoking, alcohol consumption, supplement use, physical activity energy expenditure and chronic health conditions. The second “energy-adjusted model included total energy intake as a covariate (not fitted when the energy intake was used as the outcome). ¶The odds ratios in the “Total population” column represents the odds ratio per 20-point higher PHD score on a continuous scale from logistic regression models. CI: confidence interval. mg: milligram. OR: odds ratio. P trend: P-value for trend. PHD: planetary health diet. Q: quintile. Ref: reference category. RNI: reference nutrient intake. SD: standard deviation. µg: microgram.

# Supplementary Table 12. Nutritional biomarker status of selected nutrients in adolescents aged 15-18 years in the UK National Diet and Nutrition Survey 2008-2019 (n=632).

| **Nutritional biomarker concentration** | **Total population**  **(n=632)** | **PHD Quintile 1***  **(n=121)** | **PHD Quintile 2**  **(n=117)** | **PHD Quintile 3**  **(n=127)** | **PHD Quintile 4**  **(n=126)** | **PHD Quintile 5**  **(n=131)** | **P trend†** |
| --- | --- | --- | --- | --- | --- | --- | --- |
| **Vitamin B12 (pmol/)*** | 268 (260, 277) | 255 (235, 277) | 247 (232, 263) | 276 (258, 295) | 277 (261, 294) | 282 (263, 303) | 0.009 |
| Biochemical threshold%‡ | 96.1 | 91.4 | 97.4 | 94.2 | 99.6 | 97.2 |  |
| Crude OR (95% CI) | 2.52 (0.99, 6.41)¶ | 1.00 (Ref) | 3.54 (1.23, 10.14) | 1.52 (0.47, 4.94) | 23.32 (2.66, 204.49) | 3.33 (0.86, 12.82) | 0.073 |
| Adjusted OR§ | 4.43 (1.36, 14.38)¶ | 1.00 (Ref) | 7.40 (1.57, 34.88) | 1.79 (0.34, 9.44) | 93.66 (5.79, 1,515.01) | 8.39 (1.33, 52.96) | 0.005 |
| + energy-adjusted | 4.25 (1.38, 13.05)¶ | 1.00 (Ref) | 7.52 (1.75, 32.32) | 1.83 (0.37, 9.05) | 96.93 (5.94, 1,580.54) | 8.60 (1.58, 46.81) | 0.003 |
|  |  |  |  |  |  |  |  |
| **HoloTC (pmol/L)*** | 56.2 (53.1, 59.5) | 58.6 (51.0, 67.4) | 50.1 (44.1, 57.0) | 56.8 (49.8, 64.7) | 57.8 (52.7, 63.5) | 56.4 (50.5, 63.0) | 0.769 |
| Biochemical threshold%‡ | 92.2 | 96.1 | 92.1 | 86.4 | 96.6 | 91.8 |  |
| Crude OR (95% CI) | 1.25 (0.64, 2.45)¶ | 1.00 (Ref) | 0.47 (0.07, 2.96) | 0.26 (0.04, 1.45) | 1.15 (0.16, 8.51) | 0.45 (0.08, 2.58) | 0.875 |
| Adjusted OR§ | 1.20 (0.45, 3.18)¶ | 1.00 (Ref) | 0.18 (0.01, 3.57) | 0.25 (0.02, 2.62) | 0.87 (0.06, 12.96) | 0.24 (0.02, 3.25) | 0.768 |
| + energy-adjusted | 1.63 (0.55, 4.78)¶ | 1.00 (Ref) | 0.26 (0.01, 4.79) | 0.45 (0.05, 4.12) | 1.57 (0.11, 22.96) | 0.45 (0.04, 4.82) | 0.813 |
|  |  |  |  |  |  |  |  |
| **25-OHD (nmol/L)*** | 37.9 (35.8, 40.3) | 39.3 (35.7, 43.3) | 35.6 (31.3, 40.6) | 39.9 (34.9, 45.7) | 41.8 (37.6, 46.5) | 33.9 (29.6, 38.8) | 0.430 |
| Biochemical threshold%‡ | 78.8 | 81.6 | 78.5 | 80.9 | 82.8 | 70.9 |  |
| Crude OR (95% CI) | 0.74 (0.47, 1.16)¶ | 1.00 (Ref) | 0.82 (0.36, 1.86) | 0.95 (0.42, 2.18) | 1.08 (0.50, 2.33) | 0.55 (0.25, 1.18) | 0.237 |
| Adjusted OR§ | 0.67 (0.37, 1.22)¶ | 1.00 (Ref) | 0.72 (0.26, 1.98) | 0.84 (0.29, 2.42) | 0.90 (0.31, 2.59) | 0.50 (0.17, 1.47) | 0.326 |
| + energy-adjusted | 0.70 (0.38, 1.29)¶ | 1.00 (Ref) | 0.75 (0.27, 2.12) | 0.90 (0.31, 2.63) | 0.99 (0.32, 3.08) | 0.55 (0.18, 1.65) | 0.435 |
|  |  |  |  |  |  |  |  |
| **Ferritin (µg/L)*** | 31.9 (29.0, 35.0) | 36.6 (29.4, 45.7) | 30.5 (24.6, 37.7) | 37.2 (31.2, 44.4) | 31.5 (24.6, 40.5) | 26.0 (21.8, 30.9) | 0.048 |
| Biochemical threshold%‡ | 81.9 | 82.9 | 84.3 | 88.0 | 79.4 | 75.4 |  |
| Crude OR (95% CI) | 0.62 (0.38, 1.03)¶ | 1.00 (Ref) | 1.11 (0.47, 2.65) | 1.51 (0.62, 3.69) | 0.80 (0.31, 2.03) | 0.63 (0.27, 1.47) | 0.183 |
| Adjusted OR§ | 0.65 (0.36, 1.17)¶ | 1.00 (Ref) | 1.59 (0.53, 4.78) | 2.08 (0.71, 6.13) | 1.30 (0.47, 3.61) | 0.82 (0.29, 2.35) | 0.545 |
| + energy-adjusted | 0.63 (0.35, 1.14)¶ | 1.00 (Ref) | 1.54 (0.51, 4.66) | 2.03 (0.70, 5.90) | 1.25 (0.45, 3.49) | 0.79 (0.27, 2.31) | 0.495 |
|  |  |  |  |  |  |  |  |
| **Zinc (µmol/L)*** | 14.1 (13.8, 14.3) | 14.3 (13.8, 14.8) | 14.4 (14, 14.9) | 13.8 (13.4, 14.3) | 13.8 (13.1, 14.6) | 14 (13.6, 14.5) | 0.19 |
| Beta coefficient (95% CI) | - | - | - | - | - | - |  |
| Crude | -1.04 (-3.52, 1.50) | - | - | - | - | - | 0.42 |
| Adjusted | 0.17 (-2.00, 2.39) | - | - | - | - | - | 0.88 |
| +energy-adjusted | 0.52 (-1.62, 2.70) | - | - | - | - | - | 0.64 |

* Geometric mean and 95% confidence intervals are reported. The score ranges of the five categories were <62.0 points for Q1, 62.0-69.8 points for Q2, 69.9-77.4 points for Q3, 77.5-86.2 points for Q4, and ≥86.3 points for Q5. Mean±SD was 75.8±13.9 points. Models were conducted using the survey design and sampling weights accounted for.

†P-values for trend across the quintile categories were computed by fitting the ordinal variable of the PHD quintile groups as the exposure variable. Covariates in each model corresponded to the estimation in each row (crude for estimated averages and crude or multivariable adjusted for odds ratios).

‡Those meeting or exceeding the biochemical threshold are presented as proportions for the total population and by PHD quintile.

§ Crude and multivariable-adjusted odds ratios (ORs) and corresponding 95% confidence intervals (CIs) were estimated with three models. The first adjusted model included selected covariates: age, sex, ethnicity, occupation, income, region, survey year, season, body-mass index, smoking, alcohol consumption, supplement use, physical activity energy expenditure and chronic health conditions. The second “energy-adjusted model” included total energy intake as a covariate. ¶The odds ratios in the “Total population” column represents the odds ratio per 20-point higher PHD score on a continuous scale from logistic regression models. For circulating zinc concentrations, there is no NDNS-established biomarker threshold for sufficiency, as such, the beta-coefficients and p-values per 20-point higher PHD score on a continuous scale are reported rather than odds ratios. The beta-coefficient (95%CI) was back-transformed in the following way: 100 x (exp(ln β-coefficient)-1). 25-OHD: 25-hydroxyvitamin D. β: beta coefficient. CI: confidence interval. HoloTC: holotranscobalamin. NDNS: National Diet and Nutrition Survey. OR: odds ratio. P trend: P-value for trend. PHD: planetary health diet. pmol/L: picomoles per liter. Q: quintile. Ref: reference category. SD: standard deviation. µg/L: micrograms per liter. µmol/L: micromoles per liter.

# Supplementary Table 13. Nutritional biomarker status of selected nutrients in participants aged 19-64 years in the UK National Diet and Nutrition Survey 2008-2019 (n=3105).

| **Nutritional biomarker concentration** | **Total population (n=3105)** | **PHD Quintile 1***  **(n=549)** | **PHD Quintile 2**  **(n=584)** | **PHD Quintile 3**  **(n=629)** | **PHD Quintile 4**  **(n=652)** | **PHD Quintile 5**  **(n=691)** | **P trend†** |
| --- | --- | --- | --- | --- | --- | --- | --- |
| **Vitamin B12 (pmol/)*** | 256 (252, 260) | 247 (237, 256) | 253 (245, 262) | 258 (250, 266) | 260 (252, 269) | 261 (252, 271) | 0.019 |
| Biochemical threshold%‡ | 95.4 | 96.6 | 95.5 | 95.9 | 95.4 | 94.1 |  |
| Crude OR (95% CI) | 0.77 (0.57, 1.04)¶ | 1.00 (Ref) | 0.73 (0.38, 1.42) | 0.82 (0.42, 1.59) | 0.72 (0.35, 1.46) | 0.56 (0.29, 1.05) | 0.108 |
| Adjusted OR§ | 0.71 (0.49, 1.02)¶ | 1.00 (Ref) | 0.72 (0.38, 1.36) | 0.71 (0.35, 1.46) | 0.65 (0.32, 1.32) | 0.45 (0.22, 0.95) | 0.059 |
| + energy-adjusted | 0.72 (0.49, 1.04)¶ | 1.00 (Ref) | 0.74 (0.39, 1.42) | 0.75 (0.36, 1.58) | 0.68 (0.33, 1.40) | 0.47 (0.22, 1.00) | 0.075 |
|  |  |  |  |  |  |  |  |
| **HoloTC (pmol/L)*** | 62.1 (60.5, 63.8) | 58.0 (54.5, 61.8) | 62.7 (58.7, 66.9) | 61.8 (58.6, 65.1) | 63.5 (60.1, 67.1) | 64.0 (60.6, 67.7) | 0.035 |
| Biochemical threshold%‡ | 93.5 | 91.4 | 93.8 | 94.6 | 95.9 | 92.0 |  |
| Crude OR (95% CI) | 1.02 (0.71, 1.47)¶ | 1.00 (Ref) | 1.42 (0.59, 3.45) | 1.63 (0.72, 3.72) | 2.19 (0.72, 6.66) | 1.09 (0.53, 2.24) | 0.733 |
| Adjusted OR§ | 1.18 (0.78, 1.78)¶ | 1.00 (Ref) | 1.49 (0.59, 3.77) | 2.09 (0.78, 5.61) | 3.17 (1.13, 8.95) | 1.46 (0.62, 3.43) | 0.321 |
| + energy-adjusted | 1.20 (0.78, 1.85)¶ | 1.00 (Ref) | 1.56 (0.62, 3.94) | 2.30 (0.86, 6.15) | 3.44 (1.25, 9.45) | 1.55 (0.65, 3.71) | 0.286 |
|  |  |  |  |  |  |  |  |
| **25-OHD (nmol/L)*** | 40.9 (39.7, 42.2) | 37.3 (34.7, 40.0) | 40.8 (38.7, 43.2) | 39.1 (36.3, 42.1) | 41.5 (39.3, 43.9) | 45.6 (43.6, 47.7) | <0.001 |
| Biochemical threshold%‡ | 83.2 | 76.2 | 84.4 | 80.4 | 84.4 | 89.1 |  |
| Crude OR (95% CI) | 1.49 (1.25, 1.79)¶ | 1.00 (Ref) | 1.69 (1.15, 2.49) | 1.29 (0.88, 1.88) | 1.69 (1.13, 2.54) | 2.55 (1.67, 3.91) | <0.001 |
| Adjusted OR§ | 1.44 (1.14, 1.81)¶ | 1.00 (Ref) | 1.69 (1.10, 2.60) | 1.34 (0.87, 2.09) | 1.54 (0.97, 2.44) | 2.27 (1.38, 3.74) | 0.006 |
| + energy-adjusted | 1.47 (1.16, 1.86)¶ | 1.00 (Ref) | 1.74 (1.12, 2.70) | 1.40 (0.90, 2.18) | 1.60 (1.01, 2.55) | 2.35 (1.42, 3.88) | 0.004 |
|  |  |  |  |  |  |  |  |
| **Ferritin (µg/L)*** | 65.9 (63.0, 68.9) | 75.4 (68.2, 83.3) | 66.0 (60.2, 72.5) | 71.6 (64.7, 79.4) | 64.4 (58.4, 71.0) | 55.6 (50.7, 60.8) | <0.001 |
| Biochemical threshold%‡ | 92.2 | 95.1 | 91.5 | 92.7 | 91.7 | 90.6 |  |
| Crude OR (95% CI) | 0.76 (0.61, 0.96)¶ | 1.00 (Ref) | 0.56 (0.30, 1.04) | 0.65 (0.36, 1.19) | 0.57 (0.30, 1.07) | 0.50 (0.27, 0.92) | 0.042 |
| Adjusted OR§ | 0.96 (0.74, 1.24)¶ | 1.00 (Ref) | 0.69 (0.36, 1.30) | 0.84 (0.45, 1.56) | 0.87 (0.46, 1.65) | 0.77 (0.41, 1.43) | 0.775 |
| + energy-adjusted | 0.95 (0.74, 1.23)¶ | 1.00 (Ref) | 0.68 (0.36, 1.28) | 0.83 (0.45, 1.52) | 0.86 (0.46, 1.62) | 0.76 (0.41, 1.40) | 0.745 |
|  |  |  |  |  |  |  |  |
| **Zinc (µmol/L)*** | 13.5 (13.4, 13.6) | 13.7 (13.4, 13.9) | 13.6 (13.4, 13.9) | 13.5 (13.2, 13.7) | 13.6 (13.4, 13.8) | 13.3 (13.0, 13.5) | 0.036 |
| Beta coefficient (95% CI) | - | - | - | - | - | - |  |
| Crude | -1.60 (-2.77, -0.42) | - | - | - | - | - | 0.008 |
| Adjusted | -1.27 (-2.46, -0.06) | - | - | - | - | - | 0.04 |
| +energy-adjusted | -1.20 (-2.39, -0.008) | - | - | - | - | - | 0.052 |

* Geometric mean and 95% confidence intervals are reported. The score ranges of the five categories were <62.0 points for Q1, 62.0-69.8 points for Q2, 69.9-77.4 points for Q3, 77.5-86.2 points for Q4, and ≥86.3 points for Q5. Mean±SD was 75.8±13.9 points. Models were conducted using the survey design and sampling weights accounted for.

†P-values for trend across the quintile categories were computed by fitting the ordinal variable of the PHD quintile groups as the exposure variable. Covariates in each model corresponded to the estimation in each row (crude for estimated averages and crude or multivariable adjusted for odds ratios).

‡Those meeting or exceeding the biochemical threshold are presented as proportions for the total population and by PHD quintile.

§ Crude and multivariable-adjusted odds ratios (ORs) and corresponding 95% confidence intervals (CIs) were estimated with three models. The first adjusted model included selected covariates: age, sex, ethnicity, occupation, income, region, survey year, season, body-mass index, smoking, alcohol consumption, supplement use, physical activity energy expenditure and chronic health conditions. The second “energy-adjusted model” included total energy intake as a covariate.

¶The odds ratios in the “Total population” column represents the odds ratio per 20-point higher PHD score on a continuous scale from logistic regression models.

For circulating zinc concentrations, there is no NDNS-established biomarker threshold for sufficiency, as such, the beta-coefficients and p-values per 20-point higher PHD score on a continuous scale are reported rather than odds ratios. The beta-coefficient (95%CI) was back-transformed in the following way: 100 x (exp(ln β-coefficient)-1). 25-OHD: 25-hydroxyvitamin D. β: beta coefficient. CI: confidence interval. HoloTC: holotranscobalamin. NDNS: National Diet and Nutrition Survey. OR: odds ratio. P trend: P-value for trend. PHD: planetary health diet. pmol/L: picomoles per liter. Q: quintile. Ref: reference category. SD: standard deviation. µg/L: micrograms per liter. µmol/L: micromoles per liter.

# Supplementary Table 14. Nutritional biomarker status of selected nutrients in participants aged 65 years and above in the UK National Diet and Nutrition Survey 2008-2019 (n=894).

| **Nutritional biomarker concentration** | **Total population (n=894)** | **PHD Quintile 1***  **(n=162)** | **PHD Quintile 2**  **(n=158)** | **PHD Quintile 3**  **(n=170)** | **PHD Quintile 4**  **(n=193)** | **PHD Quintile 5**  **(n=211)** | **P trend†** |
| --- | --- | --- | --- | --- | --- | --- | --- |
| **Vitamin B12 (pmol/)*** | 255 (248, 263) | 242 (229, 256) | 232 (216, 250) | 256 (238, 274) | 274 (256, 293) | 264 (249, 280) | 0.001 |
| Biochemical threshold%‡ | 94.5 | 94.1 | 90.8 | 94.7 | 97.7 | 94.4 |  |
| Crude OR (95% CI) | 1.16 (0.60, 2.22)¶ | 1.00 (Ref) | 0.62 (0.22, 1.76) | 1.13 (0.37, 3.48) | 2.73 (0.76, 9.79) | 1.07 (0.36, 3.15) | 0.308 |
| Adjusted OR§ | 0.81 (0.31, 2.13)¶ | 1.00 (Ref) | 0.84 (0.25, 2.79) | 0.97 (0.25, 3.78) | 4.01 (0.75, 21.46) | 0.77 (0.18, 3.31) | 0.939 |
| + energy-adjusted | 0.84 (0.34, 2.06)¶ | 1.00 (Ref) | 0.85 (0.24, 2.96) | 0.94 (0.23, 3.77) | 3.95 (0.73, 21.35) | 0.79 (0.19, 3.25) | 0.923 |
|  |  |  |  |  |  |  |  |
| **HoloTC (pmol/L)*** | 66.3 (63.0, 69.9) | 59.5 (53.8, 65.9) | 61.8 (53.8, 70.9) | 65.0 (56.8, 74.5) | 74.1 (67.5, 81.3) | 70.0 (64.1, 76.5) | 0.002 |
| Biochemical threshold%‡ | 94.7 | 93.8 | 91.7 | 92.5 | 98.3 | 96.2 |  |
| Crude OR (95% CI) | 1.49 (0.81, 2.74)¶ | 1.00 (Ref) | 0.72 (0.20, 2.61) | 0.82 (0.21, 3.19) | 3.80 (1.17, 12.32) | 1.65 (0.41, 6.62) | 0.112 |
| Adjusted OR§ | 1.38 (0.60, 3.21)¶ | 1.00 (Ref) | 0.76 (0.15, 3.86) | 1.10 (0.17, 6.92) | 5.28 (1.27, 21.98) | 1.40 (0.24, 7.99) | 0.227 |
| + energy-adjusted | 1.40 (0.61, 3.21)¶ | 1.00 (Ref) | 0.77 (0.15, 3.91) | 1.08 (0.18, 6.48) | 5.36 (1.33, 21.57) | 1.43 (0.25, 8.02) | 0.215 |
|  |  |  |  |  |  |  |  |
| **25-OHD (nmol/L)*** | 43.9 (42.2, 45.8) | 39.3 (35.7, 43.3) | 40.3 (37.1, 43.9) | 43.2 (39.3, 47.4) | 45.7 (42.0, 49.8) | 49.0 (45.7, 52.5) | <0.001 |
| Biochemical threshold%‡ | 85.9 | 80.0 | 84.6 | 82.6 | 88.3 | 90.9 |  |
| Crude OR (95% CI) | 1.66 (1.16, 2.38)¶ | 1.00 (Ref) | 1.37 (0.71, 2.66) | 1.19 (0.59, 2.38) | 1.89 (0.93, 3.84) | 2.50 (1.28, 4.90) | 0.006 |
| Adjusted OR§ | 1.23 (0.77, 1.96)¶ | 1.00 (Ref) | 1.06 (0.51, 2.23) | 0.71 (0.32, 1.61) | 1.38 (0.56, 3.44) | 1.36 (0.56, 3.29) | 0.430 |
| + energy-adjusted | 1.35 (0.84, 2.17)¶ | 1.00 (Ref) | 1.15 (0.55, 2.41) | 0.77 (0.34, 1.72) | 1.45 (0.59, 3.59) | 1.56 (0.66, 3.71) | 0.281 |
|  |  |  |  |  |  |  |  |
| **Ferritin (µg/L)*** | 90.0 (83.1, 97.4) | 94.9 (80.3, 112.1) | 77.5 (63.8, 94.1) | 84.2 (68.2, 104.0) | 98.9 (84.0, 116.5) | 93.1 (81.6, 106.1) | 0.403 |
| Biochemical threshold%‡ | 95.9 | 96.7 | 93.3 | 93.4 | 96.4 | 98.3 |  |
| Crude OR (95% CI) | 1.42 (0.92, 2.19)¶ | 1.00 (Ref) | 0.47 (0.16, 1.43) | 0.47 (0.15, 1.46) | 0.91 (0.25, 3.30) | 1.89 (0.53, 6.82) | 0.104 |
| Adjusted OR§ | 1.06 (0.67, 1.69)¶ | 1.00 (Ref) | 0.40 (0.09, 1.75) | 0.27 (0.08, 0.95) | 0.42 (0.10, 1.68) | 1.42 (0.33, 6.06) | 0.559 |
| + energy-adjusted | 1.06 (0.65, 1.75)¶ | 1.00 (Ref) | 0.40 (0.09, 1.69) | 0.26 (0.07, 0.94) | 0.41 (0.10, 1.69) | 1.39 (0.31, 6.15) | 0.579 |
|  |  |  |  |  |  |  |  |
| **Zinc (µmol/L)*** | 13 (12.8, 13.3) | 12.9 (12.3, 13.4) | 13.1 (12.6, 13.7) | 12.9 (12.5, 13.4) | 13.1 (12.7, 13.5) | 13.1 (12.7, 13.6) | 0.51 |
| Beta coefficient (95% CI) | - | - | - | - | - | - |  |
| Crude | 0.76 (-1.96, 3.56) | - | - | - | - | - | 0.59 |
| Adjusted | 0.89 (-1.67, 3.53) | - | - | - | - | - | 0.49 |
| +energy-adjusted | 1.15 (-1.41, 3.77) | - | - | - | - | - | 0.38 |

* Geometric mean and 95% confidence intervals are reported. The score ranges of the five categories were <62.0 points for Q1, 62.0-69.8 points for Q2, 69.9-77.4 points for Q3, 77.5-86.2 points for Q4, and ≥86.3 points for Q5. Mean±SD was 75.8±13.9 points. Models were conducted using the survey design and sampling weights accounted for.

†P-values for trend across the quintile categories were computed by fitting the ordinal variable of the PHD quintile groups as the exposure variable. Covariates in each model corresponded to the estimation in each row (crude for estimated averages and crude or multivariable adjusted for odds ratios).

‡Those meeting or exceeding the biochemical threshold are presented as proportions for the total population and by PHD quintile.

§ Crude and multivariable-adjusted odds ratios (ORs) and corresponding 95% confidence intervals (CIs) were estimated with three models. The first adjusted model included selected covariates: age, sex, ethnicity, occupation, income, region, survey year, season, body-mass index, smoking, alcohol consumption, supplement use, physical activity energy expenditure and chronic health conditions. The second “energy-adjusted model” included total energy intake as a covariate.

¶The odds ratios in the “Total population” column represents the odds ratio per 20-point higher PHD score on a continuous scale from logistic regression models.

For circulating zinc concentrations, there is no NDNS-established biomarker threshold for sufficiency, as such, the beta-coefficients and p-values per 20-point higher PHD score on a continuous scale are reported rather than odds ratios. The beta-coefficient (95%CI) was back-transformed in the following way: 100 x (exp(ln β-coefficient)-1). 25-OHD: 25-hydroxyvitamin D. β: beta coefficient. CI: confidence interval. HoloTC: holotranscobalamin. NDNS: National Diet and Nutrition Survey. OR: odds ratio. P trend: P-value for trend. PHD: planetary health diet. pmol/L: picomoles per liter. Q: quintile. Ref: reference category. SD: standard deviation. µg/L: micrograms per liter. µmol/L: micromoles per liter.

# Supplementary Table 15. Nutritional biomarker status of selected nutrients in women of reproductive age (15-49 years) in the UK National Diet and Nutrition Survey 2008-2019 (n=1466).

| **Nutritional biomarker concentration** | **Total population (n=1466)** | **PHD Quintile 1***  **(n=260)** | **PHD Quintile 2**  **(n=272)** | **PHD Quintile 3**  **(n=304)** | **PHD Quintile 4**  **(n=308)** | **PHD Quintile 5**  **(n=322)** | **P trend†** |
| --- | --- | --- | --- | --- | --- | --- | --- |
| **Vitamin B12 (pmol/)*** | 251 (245, 257) | 234 (222, 245) | 243 (231, 256) | 258 (245, 271) | 260 (247, 274) | 257 (243, 272) | 0.007 |
| Biochemical threshold%‡ | 93.5 | 92.9 | 93.2 | 93.3 | 94.3 | 93.5 |  |
| Crude OR (95% CI) | 1.04 (0.65, 1.66)¶ | 1.00 (Ref) | 1.05 (0.46, 2.39) | 1.06 (0.48, 2.35) | 1.26 (0.48, 3.31) | 1.10 (0.47, 2.58) | 0.741 |
| Adjusted OR§ | 1.01 (0.58, 1.74)¶ | 1.00 (Ref) | 0.94 (0.40, 2.23) | 1.03 (0.45, 2.38) | 1.22 (0.55, 2.73) | 0.96 (0.38, 2.45) | 0.901 |
| + energy-adjusted | 1.06 (0.58, 1.94)¶ | 1.00 (Ref) | 1.01 (0.42, 2.40) | 1.16 (0.48, 2.77) | 1.38 (0.61, 3.14) | 1.07 (0.40, 2.91) | 0.753 |
|  |  |  |  |  |  |  |  |
| **HoloTC (pmol/L)*** | 56.7 (54.4, 59.0) | 50.3 (44.9, 56.2) | 57.6 (52.0, 63.8) | 57.3 (51.8, 63.4) | 57.9 (53.6, 62.6) | 58.2 (53.5, 63.3) | 0.109 |
| Biochemical threshold%‡ | 90.3 | 81.7 | 91.4 | 88.8 | 94.8 | 91.5 |  |
| Crude OR (95% CI) | 1.41 (0.82, 2.43)¶ | 1.00 (Ref) | 2.39 (0.72, 7.85) | 1.77 (0.64, 4.94) | 4.08 (0.73, 22.92) | 2.41 (0.90, 6.45) | 0.112 |
| Adjusted OR§ | 1.67 (0.93, 2.97)¶ | 1.00 (Ref) | 2.38 (0.79, 7.19) | 2.27 (0.66, 7.82) | 5.87 (1.91, 17.97) | 3.36 (1.16, 9.72) | 0.020 |
| + energy-adjusted | 1.78 (0.96, 3.29)¶ | 1.00 (Ref) | 2.62 (0.90, 7.65) | 2.77 (0.83, 9.30) | 7.27 (2.50, 21.18) | 3.93 (1.29, 12.00) | 0.015 |
|  |  |  |  |  |  |  |  |
| **25-OHD (nmol/L)*** | 41.6 (39.9, 43.4) | 39.9 (35.9, 44.4) | 42.7 (38.9, 46.7) | 40.5 (36.9, 44.5) | 39.8 (36.5, 43.5) | 44.8 (42.0, 47.7) | 0.211 |
| Biochemical threshold%‡ | 82.7 | 79.2 | 86.3 | 82.1 | 78.2 | 86.7 |  |
| Crude OR (95% CI) | 1.14 (0.87, 1.48)¶ | 1.00 (Ref) | 1.66 (0.93, 2.96) | 1.20 (0.67, 2.17) | 0.94 (0.53, 1.69) | 1.72 (0.96, 3.06) | 0.461 |
| Adjusted OR§ | 1.33 (0.96, 1.84)¶ | 1.00 (Ref) | 1.67 (0.90, 3.11) | 1.55 (0.80, 3.01) | 1.06 (0.57, 2.01) | 2.19 (1.09, 4.39) | 0.144 |
| + energy-adjusted | 1.34 (0.96, 1.88)¶ | 1.00 (Ref) | 1.69 (0.89, 3.19) | 1.58 (0.81, 3.08) | 1.09 (0.57, 2.08) | 2.22 (1.09, 4.52) | 0.133 |
|  |  |  |  |  |  |  |  |
| **Ferritin (µg/L)*** | 31.6 (29.8, 33.5) | 32.4 (28.2, 37.3) | 33.2 (29.4, 37.5) | 33.1 (29.1, 37.7) | 30.0 (26.6, 33.8) | 30.3 (26.7, 34.3) | 0.229 |
| Biochemical threshold%‡ | 81.6 | 83.4 | 85.6 | 79.2 | 82.8 | 78.1 |  |
| Crude OR (95% CI) | 0.90 (0.70, 1.16)¶ | 1.00 (Ref) | 1.18 (0.63, 2.23) | 0.76 (0.42, 1.38) | 0.96 (0.53, 1.76) | 0.71 (0.40, 1.26) | 0.158 |
| Adjusted OR§ | 0.86 (0.65, 1.14)¶ | 1.00 (Ref) | 0.98 (0.53, 1.83) | 0.66 (0.36, 1.20) | 0.88 (0.46, 1.66) | 0.62 (0.33, 1.13) | 0.111 |
| + energy-adjusted | 0.86 (0.65, 1.13)¶ | 1.00 (Ref) | 0.98 (0.53, 1.81) | 0.64 (0.35, 1.15) | 0.85 (0.46, 1.59) | 0.60 (0.33, 1.09) | 0.090 |
|  |  |  |  |  |  |  |  |
| **Zinc (µmol/L)*** | 13.0 (12.9, 13.2) | 13.2 (12.8, 13.6) | 13.3 (12.9, 13.7) | 13.0 (12.6, 13.4) | 12.9 (12.6, 13.2) | 12.9 (12.6, 13.2) | 0.058 |
| Beta coefficient (95% CI) | - | - | - | - | - | - |  |
| Crude | -1.34 (-3.21, 0.57) | - | - | - | - | - | 0.17 |
| Adjusted | -1.76 (-3.9, 0.44) | - | - | - | - | - | 0.12 |
| +energy-adjusted | -1.54 (-3.69, 0.66) | - | - | - | - | - | 0.17 |

* Geometric mean and 95% confidence intervals are reported. The score ranges of the five categories were <62.0 points for Q1, 62.0-69.8 points for Q2, 69.9-77.4 points for Q3, 77.5-86.2 points for Q4, and ≥86.3 points for Q5. Mean±SD was 75.8±13.9 points. Models were conducted using the survey design and sampling weights accounted for.

†P-values for trend across the quintile categories were computed by fitting the ordinal variable of the PHD quintile groups as the exposure variable. Covariates in each model corresponded to the estimation in each row (crude for estimated averages and crude or multivariable adjusted for odds ratios).

‡Those meeting or exceeding the biochemical threshold are presented as proportions for the total population and by PHD quintile.

§ Crude and multivariable-adjusted odds ratios (ORs) and corresponding 95% confidence intervals (CIs) were estimated with three models. The first adjusted model included selected covariates: age, sex, ethnicity, occupation, income, region, survey year, season, body-mass index, smoking, alcohol consumption, supplement use, physical activity energy expenditure and chronic health conditions. The second “energy-adjusted model included total energy intake as a covariate

¶The odds ratios in the “Total population” column represents the odds ratio per 20-point higher PHD score on a continuous scale from logistic regression models.

For circulating zinc concentrations, there is no NDNS-established biomarker threshold for sufficiency, as such, the beta-coefficients and p-values per 20-point higher PHD score on a continuous scale are reported rather than odds ratios. The beta-coefficient (95%CI) was back-transformed in the following way: 100 x (exp(ln β-coefficient)-1).

25-OHD: 25-hydroxyvitamin D. β: beta coefficient. CI: confidence interval. HoloTC: holotranscobalamin. NDNS: National Diet and Nutrition Survey. OR: odds ratio. P trend: P-value for trend. PHD: planetary health diet. pmol/L: picomoles per liter. Q: quintile. Ref: reference category. SD: standard deviation. µg/L: micrograms per liter. µmol/L: micromoles per liter.

# References of supplementary materials

1. Bui LP, Pham TT, Wang F, Chai B, Sun Q, Hu FB, et al. Planetary Health Diet Index and risk of total and cause-specific mortality in three prospective cohorts. The American Journal of Clinical Nutrition. 2024;120(1):80-91.

2. Tong TYN, Wareham NJ, Khaw K-T, Imamura F, Forouhi NG. Prospective association of the Mediterranean diet with cardiovascular disease incidence and mortality and its population impact in a non-Mediterranean population: the EPIC-Norfolk study. BMC Medicine. 2016;14(1):135.

3. British Nutrition Foundation (BNF). Nutrition requirements. London; 2021. https://www.nutrition.org.uk/media/1z2ekndj/nutrition-requirements-update.pdf.

4. Venables MC, Roberts C, Nicholson S, Bates B, Jones KS, Ashford R, et al. Data Resource Profile: United Kingdom National Diet and Nutrition Survey Rolling Programme (2008–19). International Journal of Epidemiology. 2022;51(4):e143-e55.

5. Office for Health Improvement and Disparities. National Diet and Nutrition Survey. London: OHID; https://www.gov.uk/government/collections/national-diet-and-nutrition-survey.

6. Siemens Healthcare Diagnostics, Inc. 510(k) Summary of Safety and Effectiveness for the Atellica IM Ferritin Assay 2017 [Available from: https://www.accessdata.fda.gov/cdrh_docs/pdf17/K171642.pdf.

7. UCSF Clinical Labs-Chemistry. Vitamin B12. ADVIA Centaur System [Available from: https://ptacts.uspto.gov/ptacts/public-informations/petitions/1459793/download-documents?artifactId=g0FwHQDqV7hJVKMiRAfmJs1uu_x7GnnT9a3XLfjdW6Wq2RpHrdhGits.

8. IBL International GmbH. Active-B12 (Holotranscobalamin) ELISA. Instructions for Use [Available from: https://ibl-international.com/media/mageworx/downloads/attachment/file/3/0/30221798_ifu_ww_en_active_b12_elisa_2023-06_sym9.pdf.

9. Ersfeld DL, Rao DS, Body JJ, Sackrison JL, Jr., Miller AB, Parikh N, et al. Analytical and clinical validation of the 25 OH vitamin D assay for the LIAISON automated analyzer. Clin Biochem. 2004;37(10):867-74.

10. Vislisel JM, Schafer FQ, Buettner GR. A simple and sensitive assay for ascorbate using a plate reader. Anal Biochem. 2007;365(1):31-9.

11. Piraner O, Jones RL. Urine gross alpha/beta bioassay method development using liquid scintillation counting techniques. J Radioanal Nucl Chem. 2021;327(1):513-23.

12. Kalmbach R, Paul L, Selhub J. Determination of unmetabolized folic acid in human plasma using affinity HPLC. Am J Clin Nutr. 2011;94(1):343s-7s.
